# Circ-PTPDC1 promotes the Progression of Gastric Cancer through Sponging Mir-139-3p by Regulating ELK1 and Functions as a Prognostic Biomarker

**DOI:** 10.7150/ijbs.62732

**Published:** 2021-10-17

**Authors:** Zhouxiao Li, Ye Cheng, Kai Fu, Qiaowei Lin, Tianyu Zhao, Weiwei Tang, Lei Xi, Lulu Sheng, Hao Zhang, Yangbai Sun

**Affiliations:** 1 Department of Musculoskeletal Oncology, Fudan University Shanghai Cancer Center, Shanghai Medical College, Fudan University, Shanghai, 200032, China.; 2 Division of Hand, Plastic and Aesthetic Surgery, University Hospital, LMU Munich, Pettenkoferstrasse 8a, 80336 Munich, Germany.; 3 Department of General Surgery, Nanjing Drum Tower Hospital, The Affiliated Hospital of Nanjing University Medical School, Nanjing, Jiangsu, China; 4 Department of General Surgery, Nanjing First Hospital, The Affiliated Nanjing Hospital of Nanjing Medical University, Nanjing, Jiangsu, China.; 5 Institute and Clinic for Occupational, Social and Environmental Medicine, LMU University Hospital Munich; 6 Jiangsu Province Hospital, The first affiliated hospital of Nanjing Medical University, Nanjing, Jiangsu, China; 7 Department of Emergency Medicine, Shanghai Jiao Tong University Affiliated Six People's Hospital, Shanghai, China.; 8 Department of Orthopedic Oncology, Shanghai Changzheng Hospital, Second Military Medical University, Shanghai, China.

**Keywords:** gastric cancer, circ-PTPDC1, miR-139-3p, ELK, prognosis

## Abstract

Circular RNAs (circRNAs) is a novel class of non-coding RNAs resulting from the non-canonical splicing of linear pre-mRNAs. However, the role of circRNAs in gastric cancer (GC) remains indistinct. This study aims to explore their potential modulation in GC and its prognostic value. We first screen for circRNA expression patterns in GC through GC and adjacent noncancerous tissues by microarray. Based on the bioinformatics analysis of the microarray data, we screened out a novel circRNA, circ-PTPDC1. Then we demonstrated that circ-PTPDC1 was up-regulated in GC cells, tissues, and serum. Its overexpression was positively correlated with age, invasion depth, advanced clinical stages, and worse survival in patients with GC. We further revealed that circ-PTPDC1 promotes the proliferation, migration, and invasion of GC cell lines via sponging miR-139-3p by regulating ELK1. Importantly, we identified that circ-PTPDC1 promotes tumor upgrowth and metabasis in vivo. Additionally, we established its prognostic prediction model based on the follow-up data of the patients. Our study revealed a novel regulatory mechanism and a comprehensive landscape of circ-PTPDC1 in GC, suggesting that circ-PTPDC1 has the potential to be a biomarker for early detection and prognostic prediction of GC.

## Introduction

Gastric cancer (GC), as the world's third largest tumor, poses a great threat to human health 1. In China, GC ranks second in the incidence of cancer with an estimate of 679,100 new cases and 498,000 deaths in 2015 2. The 5-year overall survival (OS) of GC in most countries is still less than 30%, despite advances in surgical techniques and combined chemotherapy strategies. As there is no specific bio indicator before GC progresses to an advanced stage 3, it is necessary to explore molecular characters of GC for the sake of developing a rational approach for early detection of the disease.

Circular RNAs (circRNAs) are considered to be produced by non-canonical splicing of linear pre-mRNAs. CircRNAs exist broadly in virus-like infectious particles, such as viroid, circular satellite viruses, or the hepatitis delta virus 4-5. It seems that the results of false RNA splicing and the key roles and functions of circRNAs have not been well understood yet. Previous evidence suggested circRNA as a special type of endogenous non-coding RNA derived from unconventional forms of alternative splicing, originating from exons (exon circRNA, ecircRNA), introns (introns circRNA, ciRNA) or both 6. Most circRNAs are formed by exons, and their formation influences their cyclization characteristics 7. Previous research also made great efforts to determine the possible role of circRNAs regulating transcription and pathways by manipulating microRNAs (miRNAs) 8-9. It reports that circRNAs can function as competitive endogenous RNAs (ceRNAs), which regulate the protein expression via competition for shared microRNAs. Their roles in mammals are mainly as miRNA sponges or ceRNAs 10-11. Nonetheless, abundant circRNAs that can function as ceRNAs may not yet be discovered because of our limited understanding of circRNAs. Meanwhile, plentiful discoveries have permanently altered our perspectives toward the function and role of circRNAs in carcinomas, especially in carcinogenesis and cancer progression.

To date, several endogenous circRNAs have been found in mammalian cells, and they are highly abundant and evolutionarily conserved during tumorigenesis 12-14. In addition, several circRNAs are considered to be responsible for the malignant biological behavior of cancer cells15. With the deepening of relative research, circRNA has been found to produce a crucial effect in the occurrence and development of various malignant tumors and could serve as a kind of novel tumor marker. It was found in our previous study 16 that circ-PSMC3 was significantly down-regulated in GC tissues in comparison with that in the corresponding normal tissues, plasma and GES-1 cell line. Clinicopathological features showed that circ-PSMC3 level in GC tissues was negatively correlated with TNM stage and shorter OS. In addition, circ-PSMC3 acted as a sponge of miR-296-5p to regulate the expression of phosphatase and tensin homolog, and further suppressed the tumorigenesis of GC cells 16. Zhang et al. 17 found that circ-LARP4 low expressed in GC tissues and suppressed GC cells by absorbing miR-424 and aiming LATS1, which act as a specialty prognostic element for OS of patients with GC 17. All these findings demonstrate that circRNAs could function as miRNA sponges to modulate tumor progression. However, the role of circRNAs in GC still remains largely undefined at present and their features and functions require further investigation.

In the current study, we used human circRNA microarray analysis to screen for the circRNA expression profiles in GC tissues and precancerous tissues from 6 GC patients, from which we identified a circRNA termed as circ-PTPDC1 originated from PTPDC1 gene. Based on the bioinformatic analysis results, a series of functional verification experiments were conducted to explore the role of circ-PTPDC1 in the development of GC. The results showed that circ-PTPDC1 promoted proliferation and migration of GC cells by sponging miR-139-3p, thereby promoting transcription of E26 transformation-specific Like-1 protein (ELK1), and further improved the tumorigenesis of GC cells. Follow-up data showed that patients with higher circ-PTPDC1 expression levels had shorter disease--free survival (DFS) and OS, indicating that circ-PTPDC1 can be used as a biomarker for diagnosis of GC and a potential target for treatment of GC.

## Results

### CircRNA expression profiles in GC progression

**Figure 1:**
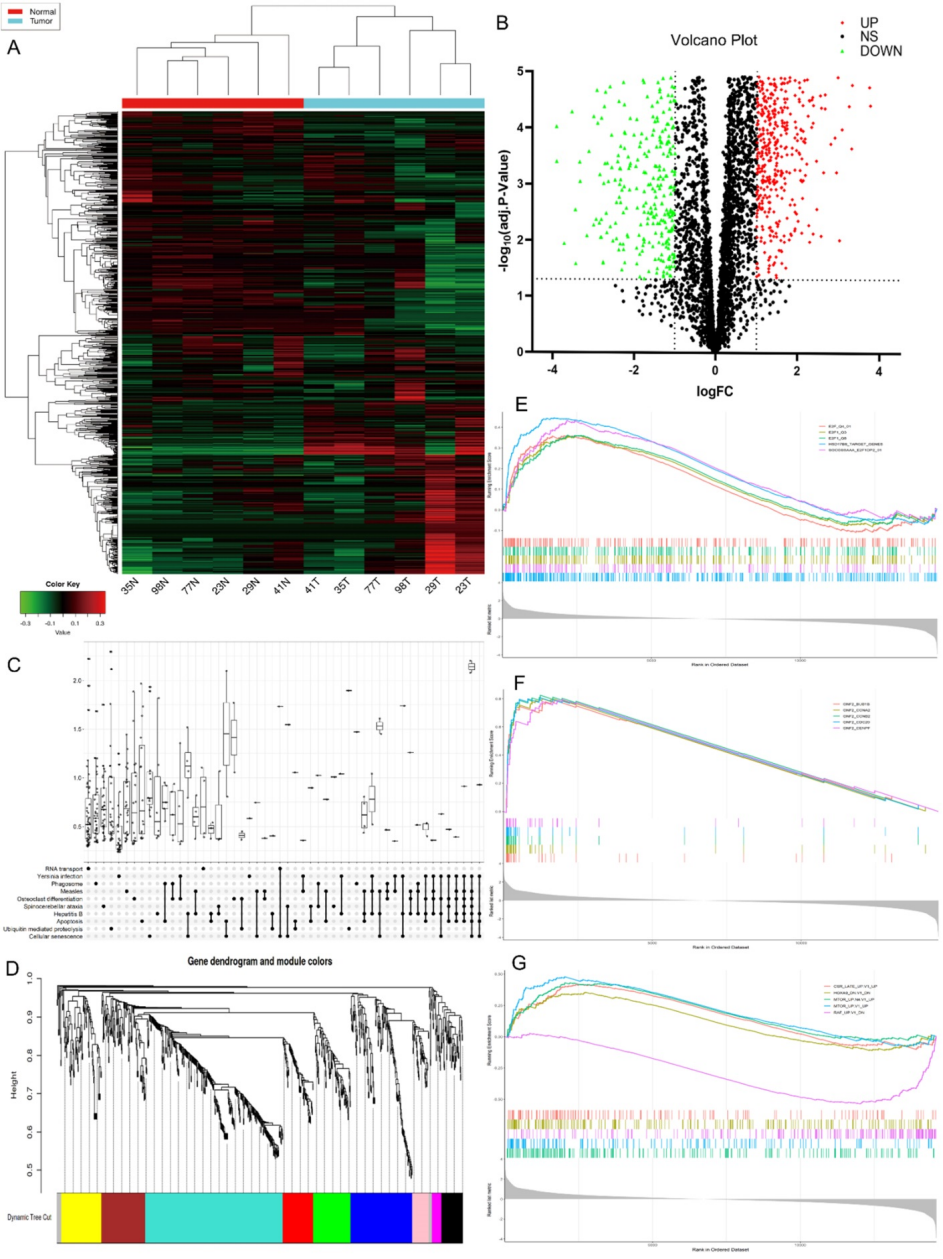
** Profiling of circular RNAs in gastric cancer (GC) microarray data sets and the bioinformatic analysis of microarray data. (A)** Heatmap and hierarchical cluster analysis, based on the expression levels of circRNAs. CircRNA microarray expression profiles from six pairs of GC tissue samples (T-tumor) and adjacent noncancerous tissues samples (N-Normal). Each column means the expression profile of a tissue specimen, and each row corresponds to a circRNA. “Red” signifies higher expression level, and “green” signifies lower expression level. **(B)** Volcano plot shows the upregulated and downregulated circRNAs in GC *vs.* control. The vertical dot lines correspond to 2.0‑fold increased or decreased expression, and the horizontal dot line represents P<0.05. **(C)** KEGG classification in GC *vs.* Control, P<0.05. **(D)** Weighted gene co-expression network analysis (WGCNA) was performed to construct a scale-free network of the CircRNA's target gene, and the whole-genome genes were assigned to seven different modules. **(E-F)** Gene Set Enrichment Analysis (GSEA) analysis of the microarray data. E. The five most significant regulatory targets. F. The five most significant cancer gene neighborhoods and cancer modules. **(G)** The five most significant gene signatures.

The high-throughput human circRNA microarray was conducted using the tissue samples from six GC patients. Six GC tissues and their matching adjacent noncancerous tissues were collected, their clinicopathological features were displayed in **Table S1**. The human circRNA microarray was used to screen for dysregulated circRNA. A total of 921 circRNAs were differentially expressed in GC tissues versus non-GC tissues among all detected candidate circRNAs according to the t-test. Among them, 391 were significantly up-regulated and 530 were significantly down-regulated in GC tissues. The data are presented in the format of heat maps **(Fig. 1A)** and volcano plots** (Fig. 1B)** in GC versus non-GC group.

### Bioinformatics prediction analysis

**Figure 2:**
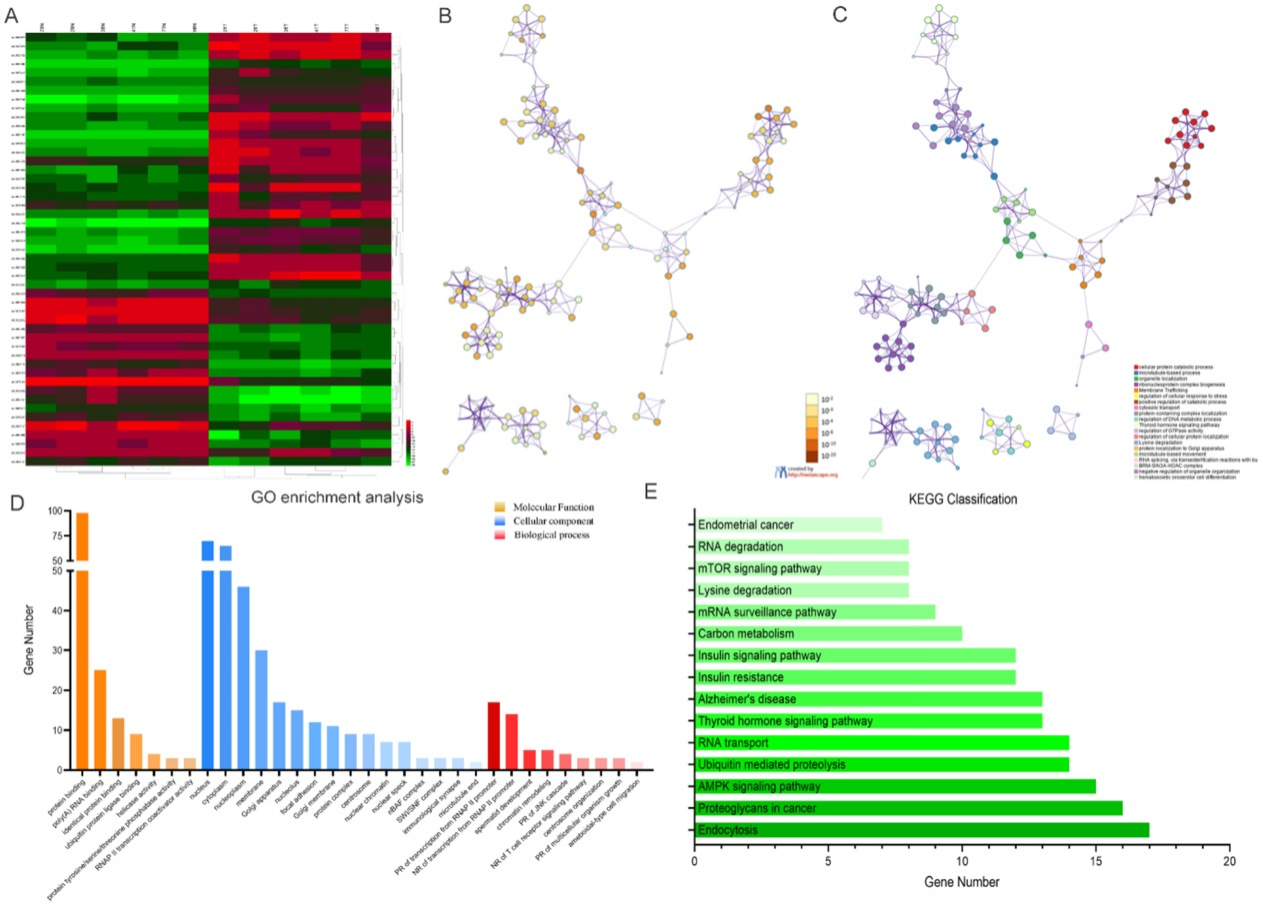
** Profiling of the top 50 significant differentially expressed circular RNAs in GC microarray data sets and the correspondent bioinformatics analysis. (A)** Heat map showing the top 50 circRNAs with the biggest absolute log_2_FC values. **(B)** The network of enriched terms colored by p-value; terms containing more genes tend to have smaller p-values. **(C)** The network of enriched terms colored by cluster ID; nodes that share the same cluster ID are typically close to each other. **(D)** GO enrichment analysis of the circRNAs' target genes. **(E)** Results of KEGG analysis of the circRNAs' target genes.

Kyoto Encyclopedia of Genes and Genomes (KEGG) analysis suggested that these differentially expressed circRNAs were primarily involved in RNA transport, Yersinia infection, apoptosis and other biological processes **(Fig. 1C).** Weighted Gene Correlation Network Analysis (WGCNA) showed that these differentially expressed circRNAs were mainly assigned to seven different modules **(Fig. 1D).** Gene Set Enrichment Analysis (GSEA) revealed five most relevant signatures of these circRNAs in regulatory target gene sets (C3), computational gene sets (C4) and oncogenic gene sets (C6) **(Fig.1E-G).** A total of 50 up-regulated and down-regulated circRNAs were selected from the high to low based on the difference in multiple expression between cancer and adjacent noncancerous tissues, based on which a heat map was worked out **(Fig. 2A).** Enrichment analysis by Metascape and GO, KEGG showed that these differentially expressed circRNAs were associated with several important physiological processes, cellular components, molecular functions and critical signaling pathways **(Fig. 2B-E).**

### The biological structure and abundance of circ-PTPDC1 in GC

**Figure 3:**
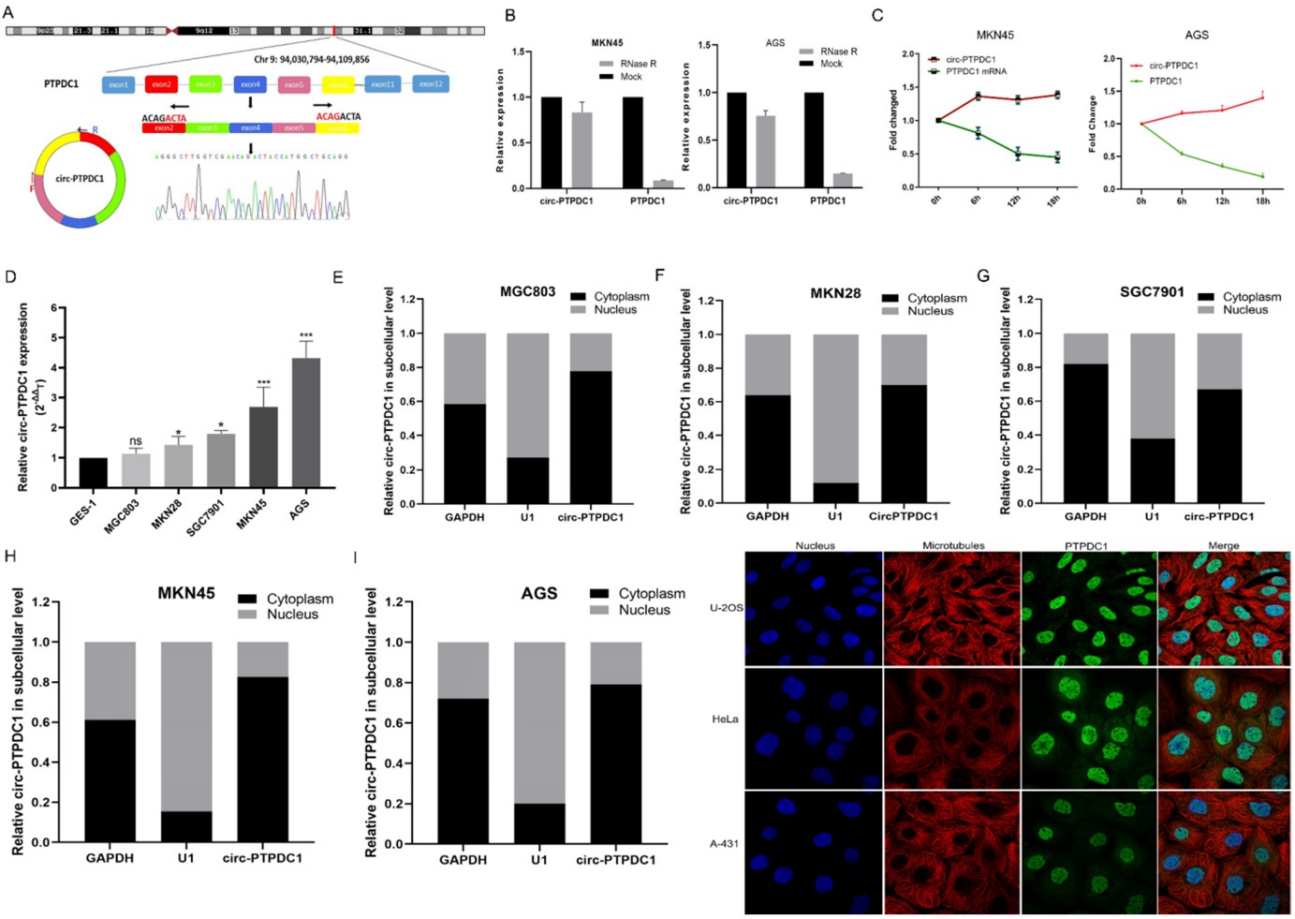
** The abundance of circ-PTPDC1 in GC cells. (A)** Schematics showing that circ-PTPDC1 was derived from the exons 2-6 of PTPDC1 gene. The amplified products were plugged into a T-vector for Sanger sequencing to ascertain their total length. **(B)** qRT-PCR for the abundance of circ-PTPDC1 and PTPDC1 mRNA in two GC cells treated with RNase R. Compared with linear mRNA, circRNA had stronger tolerance to RNase R. **(C)** qRT-PCR for the abundance of circ-PTPDC1 and PTPDC1 mRNA in GC cells treated with actinomycin. **(D)** Relative expression of circ-PTPDC1 in GC cell lines (AGS, MKN28, SGC7901, MKN45, MGC803) and human gastric epithelial cell line (GES-1). **(E-I)** qRT-PCR analysis of circ-PTPDC1 expression levels in different subcellular fractions in GC cells. GAPDH and small nuclear RNA U1 were utilized as control of cytoplasm and nucleus, respectively. **(J)** The Human Protein Atlas database revealed that PTPDC1 was colocalized with microtubule proteins in the cytoplasm of U-2 OS, HeLa and A-431 cells.

We selected a total of 10 circRNAs (top 5 high expression in tumor and top 5 low expression in tumor) according to the multiple fold difference between GC plasmas expression and normal control, and then used qRT-PCR to verify the findings in a small sample of plasmas. Among them, the fold change of circ-0008577 expression level between GC tissues and their matched pericarcinomatous tissue is 4.93, which is a relative high fold change. Results showed that circ-PTPDC1 (hsa_circ_0008577 is in gene symbol PTPDC1 and thus we named it as circ-PTPDC1) has a remarkable higher expression in GC plasmas in comparison with normal controls **(Fig. S1).** Investigation of the mechanism underlying circ-PTPDC1 formation showed that circ-PTPDC1 was derived from the exons 2,3,4,5 and 6 of the gene PTPDC1 and its expression is highly positively correlated with PTPDC1 expression level **(Fig. 3A).** Resistance to digestion with RNase R exonuclease and actinomycin D further demonstrated that circ-PTPDC1 was a stable circRNA **(Fig. 3B-C).** To explore the function of circ-PTPDC1 in GC, we determined the expression levels in five GC cells (AGS, MKN28, SGC7901, MKN45 and MGC803) and human gastric epithelial cell (GES-1) and found that circ-PTPDC1 expression was obviously higher in four GC cells and higher in AGS cells without significant difference **(Fig. 3D).** Besides, the nuclear and cytoplasmic separation experiment showed that circ-PTPDC1 localized in both the nucleus and cytoplasm but primarily in the cytoplasm **(Fig. 3E-I).** According to the Human Protein Atlas database, PTPDC1 was also located in the cytoplasm where microtubule proteins were present in U-2 OS, Hela and A-432 cells **(Fig. 3J).** This provides the most basic premise for follow-up research of the action mechanism of circ-PTPDC1.

### Circ-PTPDC1 is over-expressed in GC tissues and plasma samples

**Figure 4:**
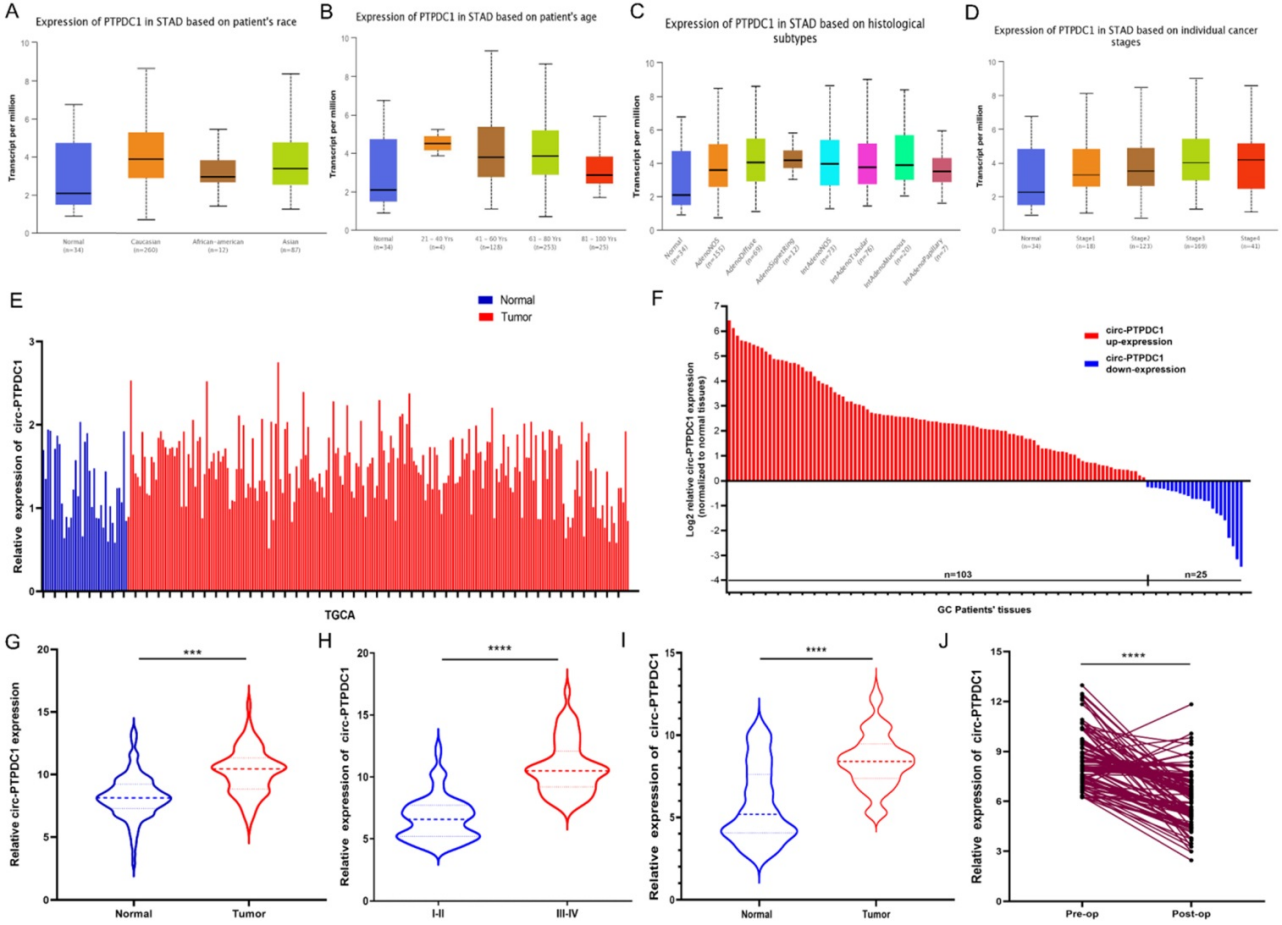
** circ-PTPDC1 (Hsa_circ_008877) was up-regulated in gastric cancer (GC) tissues. (A-D)** PTPDC1 expression is correlated with race, age, subtype and stages in GC patients. *P<0.05; **P<0.01.** (E)** Relative expression of PTPDC1 in GC *vs.* normal tissues was analyzed using TCGA data. **(F-G)** circ-PTPDC1 expression level in 128 pairs of GC tissues and matched adjacent noncancerous tissues was examined by qRT-PCR. circ-PTPDC1 was obviously up-regulated in GC tissues. **(H)** circ-PTPDC1 expression was higher in tumor tissues with III/IV stages than I/II stages. **(I)** Plasma circ-PTPDC1 was obviously up-regulated in 128 GC patients as compared with that in 128 healthy controls. **(J)** Plasm circ-PTPDC1 expression level was significantly lower post-op than that pre-op in GC patients. ***p<0.001, ****p<0.0001.

We confirmed the expression of PTPDC1 in normal and tumor tissue with UALCAN and TCGA portal firstly. The results of subgroup analysis showed the PTPDC1 expression in tissues of GC patients was significantly correlated with individual cancer race, age, subtypes, and stages **(Fig. 4A-D).** TCGA data showed that PTPDC1 expression was up regulated in the tumor tissues contrasted with expression in normal tissues **(Fig. 4E).** To validate the analysis finding, we examined and quantified the expression of circ-PTPDC1 by qRT-PCR in 128 paired clinical GC tissues and matched non-tumor tissues from GC patients, the sub-pathological types of the selected samples was shown in **Table S2**. The results showed that the expression of circ-PTPDC1 in the GC tissues was significantly lower than that in the adjacent noncancerous tissues. In line with the expression level of circ-PTPDC1 in cancer and pericarcinomatous tissues, we divided it into high-expression and low- expression group. The circ-PTPDC1 level was found to be elevated in the tumor tissues of 103 of these patients (*p*<0.001) **(Fig. 4E-F).** The average expression of circ-PTPDC1 in gastric cancer and adjacent tissues is shown in **Fig. 4G.** In addition, as shown in **Fig. 4H**, the circ-PTPDC1 expression level was significantly higher in more advanced tumors according to the patients' documents. The same trend was observed in circ-PTPDC1 expression in the plasma samples of the patient **(Fig. 4I)**. Besides, the plasma expression level of circ-PTPDC1 was reduced markedly post-operation versus pre-operation **(Fig. 4J).** Therefore, we selected circ-PTPDC1 as a candidate target for further study.

### Circ-PTPDC1 is associated with clinicopathological parameters of GC patients

The correlation between circ-PTPDC1 up-regulation and the clinicopathological features was analyzed in the 128 GC patients. As shown in **Table 1**, circ-PTPDC1 level was not correlated with gender, location, size, differentiation, or lymphatic metastasis in GC patients. However, the up-expression of circ-PTPDC1 was positively correlated with age, T stage and clinical stage (*p*<0.05) **(Table 1).** In addition, univariate and multivariate analysis indicated that circ-PTPDC1 expression level, TNM stage and invasion depth were independent prognostic indicators of OS and DFS in GC patients **(Table 2).**

### Circ-PTPDC1 expression level alters proliferation, invasion and migration of GC cells

**Figure 5:**
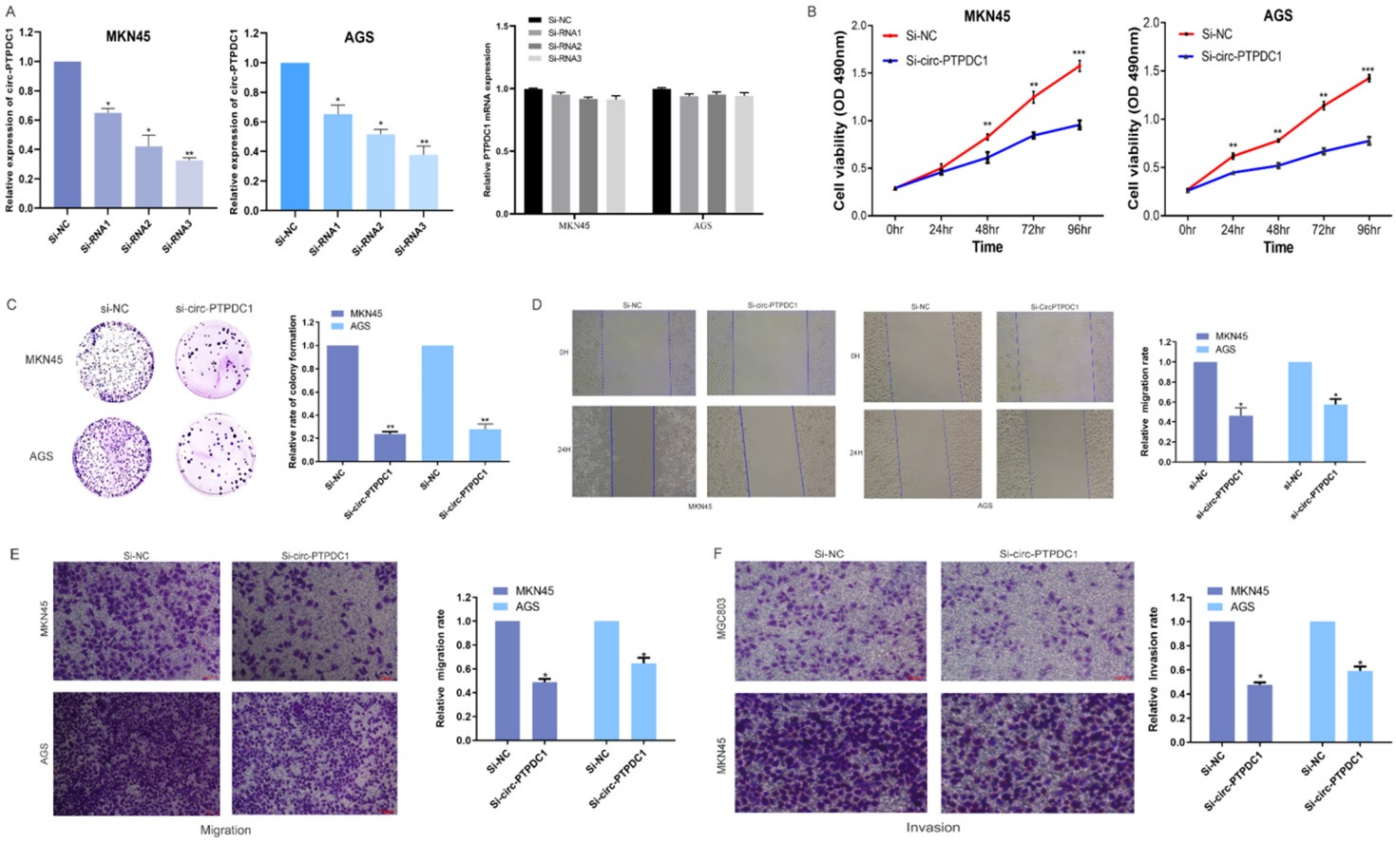
** Altered proliferation, invasion and migration of GC cells by circ-PTPDC1 expression level. (A)** Relative expression of circ-PTPDC1 and PTPDC1 mRNA in MKN45 and AGS cells transfected with siRNAs. **(B-C)** CCK-8 assay and colony formation assay demonstrated that circ-PTPDC1 knockdown significantly inhibited cell proliferation in GC cells. *P < 0.05, **P < 0.01. **(D)** Inhibition of circ-PTPDC1 produced a lower scratch closure rate than that treated with si-NC in GC cells. **(E-F)** Cell migration and invasion assays were conducted in cells transfected with circ-PTPDC1 and miR-139-3p mimics through transwell cabinet with or without Matrigel respectively. * P<0.05, ** P<0.01, *** P<0.001.

To further investigate the role of circ-PTPDC1 in the progression of GC, GC cell lines MKN45 and AGS, which exhibited the highest level of circ-PTPDC1 expression, were selected to the transfected si-RNAs. The transfection efficiency was evaluated by three si-circ-PTPDC1s and si-NC with the Lipofectamine 2000 transfection reagent. As indicated in **Fig. 5A,** the knockout effect of si-RNA1, si-RNA2 and si-RNA3 was significantly higher than that of si-NC. The CCK-8 assay was performed to generate curves of cell upgrowth over 96 h and the results showed that circ-PTPDC1 downregulation inhibited the proliferation of MKN45 and AGS markedly **(Fig. 5B).** The result of colony formation assay showed that the number of MKN45 and AGS cell colonies was decreased significantly after transfection with si-circ-PTPDC1 **(Fig. 5C).** Using the GC cell line MKN45 and AGS, cell scratches were created at 0 and 24 h after cell interference and capture of cell image. The confluent monolayer of the cultured GC cells demonstrated that suppression of circ-PTPDC1 by si-circPTPDC1 reduced the scratch closure rate as compared with control cells treated with si-NC **(Fig. 5D).** Next, the transwell assay also demonstrated that the migratory ability of the MKN45 and AGS cell lines was significantly inhibited after knocking down circ-PTPDC1** (Fig. 5E).** Then, the effect of circ-PTPDC1 on the invasion behavior of GC cell lines was assessed, manifesting that the invasive ability in AGS and MKN45 cells was significantly decreased in Matrigel substrate after the suppression of circ-PTPDC1 **(Fig. 5F).**

### Circ-PTPDC1 serves as a sponge for miR-139-3p and miR-139-3p suppresses the proliferation, migration and invasion of GC cells

**Figure 6:**
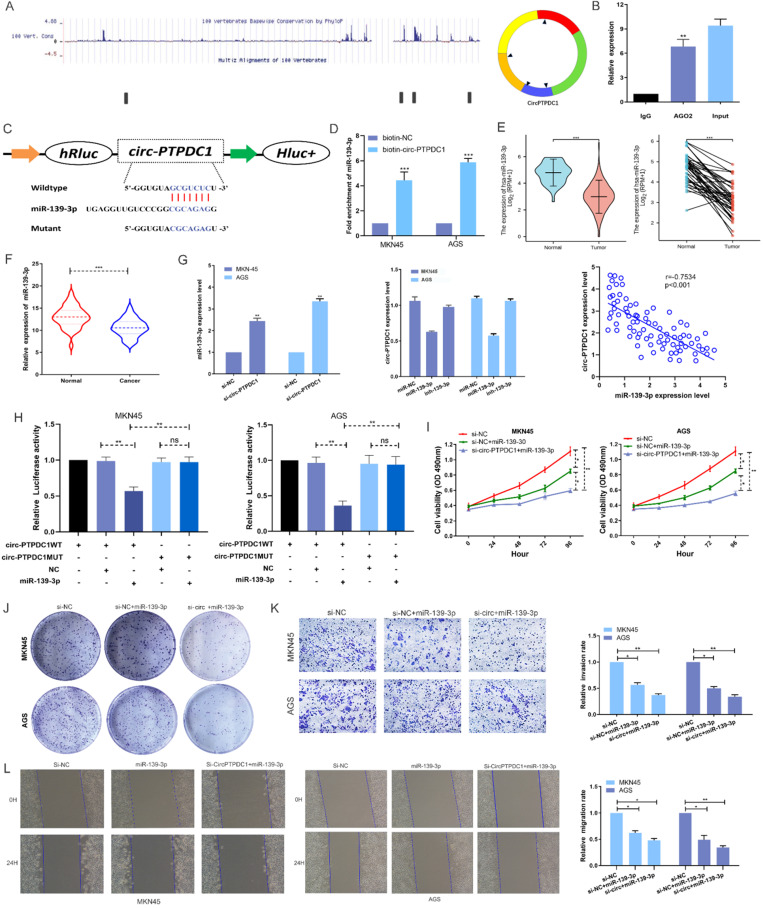
** circ-PTPDC1 is a molecular sponge of miR-139-3p and miR-139-3p suppresses the proliferation, migration and invasion of GC cells. (A)** According to the high-throughput sequencing data of doRiNA, the circ-PTPDC1 region contains a high proportion of AGO2. **(B)** RIP assays were conducted to validate whether circ-PTPDC1 could interact with miR-539 in GC cells. **(C)** A complementary binding site of circ-PTPDC1 and miR-139-3p was predicted via circinteractome and circbase online database. **(D)** The results of biotin-coupled probe pull-down assay showed that miR-139-3p and circ-PTPDC1 were detected in the circ-PTPDC1 pulled-down pellet compared with the control group. **(E)** The expression level of miR-139-3p in normal and tumor tissues in TGCA unpaired and paired samples miRNAseq databases. **(F)** miR-139-3p expression levels in tumorous tissues and adjacent noncancerous tissues of 128 GC patients. **(G)** circ-PTPDC1 expression levels in cells transfected with miR-296-5p mimics or inhibitor. miR-139-3p expression levels after transfection with si-NC or si-circ-PTPDC1 in GC cells. Spearman's correlation analysis revealed the relationship between circ-PTPDC1 expression and miR-139-3p expression in tumor tissues. **(H)** miR-139-3p mimics reduced the relative luciferase expression in circ-PTPDC1-WT compared with NC in GC cells. **(I)** The proliferation ability of cells transfected with si-circ-PTPDC11 + miR-139-3p mimics was significantly inhibited in comparision with cells transfected with miR-139-3p in GC cells. **(J)** Cell colony-formation ability was examined in GC cells transfected with miR-139-3p and co-transfected with si-circ-PTPDC1 and miR-139-3p. **(K)** Cell invasion assays were performed in GC cells transfected with circ-PTPDC1 and miR-139-3p through transwell cabinet with Matrigel. **(L)** Cell motility was detected by wound healing experiment in GC cells transfected with miR-139-3p and co-transfected with si-circ-PTPDC1 and miR-139-3p. * P<0.05, ** P<0.01, *** P<0.001.

To investigate the possibility of circ-PTPDC1 to bind to miRNAs, sequencing data available from doRiNA showed that circ-PTPDC1 had a high degree of AGO2 occupancy **(Fig. 6A).** The subsequent RNA immunoprecipitation (RIP) experiment showed that circ-PTPDC1 had a higher degree of inhibition in Ago2 immunoprecipitation compared with IgG immunoprecipitation, implying that circ-PTPDC1 could combine with miRNAs **(Fig. 6B).** To identify which miRNAs could bind to circ-PTPDC1, we used circinteractome, starbase and Targetscan to perform bio-informatics analysis. A total of 23 miRNAs and corresponding target mRNAs were predicted to have an interaction with circ-PTPDC1, and only one miRNA, miR-139-3p was identified, which also had a relatively higher context score percentile **(Figure S2).** As displayed in **Fig. 6C**, miR-139-3p possessed a potential complementary binding site for circ-PTPDC1 and was selected as a candidate target for it. The biotin-coupled probe pull-down assay was performed in order to confirm the prediction. The result indicated that miR-139-3p and circ-PTPDC1 were present in the circ-PTPDC1 pull-down pellet compared with the control group **(Fig. 6D).** We then detected the miR-139-3p expression level in TGCA unpaired and paired miRseq database, the results **(Fig. 6E)** showed that miR-139-3p is significant lower in GC tissues, which are consistent with our results via qRT-PCR analysis in tumorous tissues and adjacent noncancerous tissues of 128 GC patients **(Fig. 6F).** qRT-PCR further confirmed that circ-PTPDC1 knockdown could increase the miR-139-3p level and miR-139-3p suppresses circ-PTPDC1 level. Notably, Spearman's correlation analysis showed a significant negative correlation between circ-PTPDC1 expression and miR-139-3p expression in the cancerous tissues **(Fig. 6G).** To validate the direct binding of circ-PTPDC1 and miR-139-3p, luciferase reporter assays were carried out. As evident from **Fig. 6H**, transfection of miR-139-3p mimics induced a big fall in luciferase activity of the reporter vectors carrying wild-type circ-PTPDC1 as compared with the negative control, however, luciferase activity did not change significantly in the mutant group. We next explored the function of miR-139-3p in GC, as shown in **Fig. 6I and Fig. 6J**, the proliferative capacity of cells treated si-circ-PTPDC1 + miR-139-3p or miR-139-3p in GC cells was significantly suppressed in comparison to those treated with si-NC. The transwell assay demonstrated that the invasion ability of GC cells was inhibited by miR-139-3p and si-circ-PTPDC1 **(Fig. 6K).** Scratch wound assay showed that the migration rate of GC cells transfected or co-transfected with miR-139-3p and si-circ-PTPDC1 was significantly lower than that of GC cells transfected with si-NC **(Fig. 6L).** Collectively, these results indicate that circ-PTPDC1 acted as a molecular sponge of miR-139-3p and miR-139-3p suppresses the proliferation, migration and invasion of GC cells.

### Circ-PTPDC1 directly binds to miR-139-3p to further target ELK1

**Figure 7:**
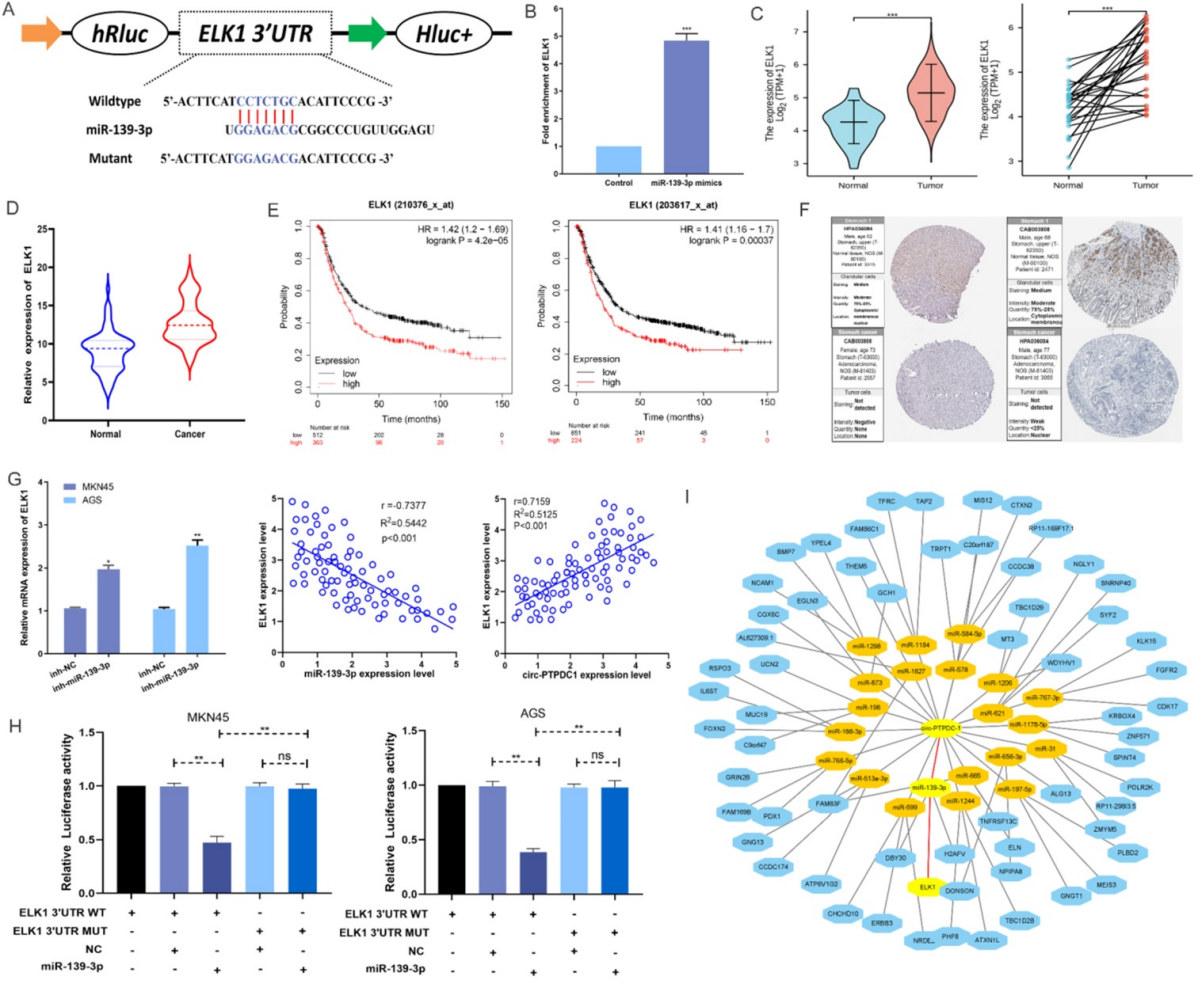
** MiR-139-3p directly target ELK1 in GC. (A)** A complementary binding site of miR-139-3p and ELK1 was predicted via Targetscan and RPIseq online database. **(B)** RIP assays revealed the interactive expression of miR-139-3p and the mRNA expression of ELK. **(C)** The expression level of ELK1 in normal and tumor tissues in TGCA unpaired and paired samples RNAseq databases. **(D)** The mRNA of ELK1 expression levels in tumorous tissues and adjacent noncancerous tissues of 128 GC patients. **(E)** High expression of ELK1 was positively correlated with poor OS in GC patients. **(F)** ELK1 staining in gastric normal tissues and GC tissues. **(G)** The mRNA expression level of ELK1 in GC cells transfected with inhibitor-NC and miR-139-3p inhibitor. We carried out a Spearman's correlation analysis to appraise the expression relationship between ELK1 and circ-PTPDC1. **(H)** miR-139-3p mimics decreased the relative luciferase expression in ELK1-WT compared with NC in GC cells. **(I)** A network showed the miRNAs and proteins associated with circ-PTPDC1.

According to MIRDB database prediction, ELK1 is the target protein of miR-139-3p with the highest score **(Table S3).** As shown in** Fig. 7A**, ELK1 mRNA 3′ UTR had a potential complementary binding site for miR-139-3p and was selected as the candidate target of miR-139-3p. RIP assays demonstrated that more ELK1 mRNA was enriched after miR-139-3p over-expression treatment compared with control treatment, suggesting that miR-139-3p could interact with ELK1 **(Fig. 7B).** The ELK1 expression is dramatically higher in TGCA paired and unpaired RNAseq database **(Fig. 7C)**, subsequent qRT-PCR confirmed that the expression level of ELK1 mRNA in the tumorous tissues of 128 GC patients was noticeably higher than that in the correspondent adjacent noncancerous tissues** (Fig. 7D)**. Besides, the analysis results in Kaplan-Meier Plotter indicated that patients with higher ELK1 expression had higher recurrence rates as well as shorter and OS **(Fig. 7E)**. Further Analysis of Human Protein Atlas data indicated that ELK1 staining is stronger in GC tissue than in adjacent noncancerous tissues **(Fig. 7F).** In addition, we found that miR-139-3p significantly reduced the ELK1 mRNA level in GC cells. The Spearman's correlation analysis showed a significant negative correlation between ELK1 expression and miR-139-3p expression and a significant positive correlation between ELK1 expression and circ-PTPDC1 expression in the cancerous tissues **(Fig. 7G).** This interaction was confirmed by luciferase reporter assays, showing that the activity of a luciferase reporter was reduced significantly after miR-139-3p over-expression as compared with NC. The inhibition of miR-139-3p increased the luciferase activity in comparison with NC with wild-type ELK1 sequence. However, these effects disappeared with mutated binding sites of miR-139-3p **(Fig. 7H).** A network diagram was drawn, showing that the main potential miRNAs and their target proteins were associated with circ-PTPDC1 **(Fig. 7I).**

### Circ-PTPDC1 promotes the proliferation, migration and invasion of GC by sponging miR-139-3p to regulate ELK1

**Figure 8:**
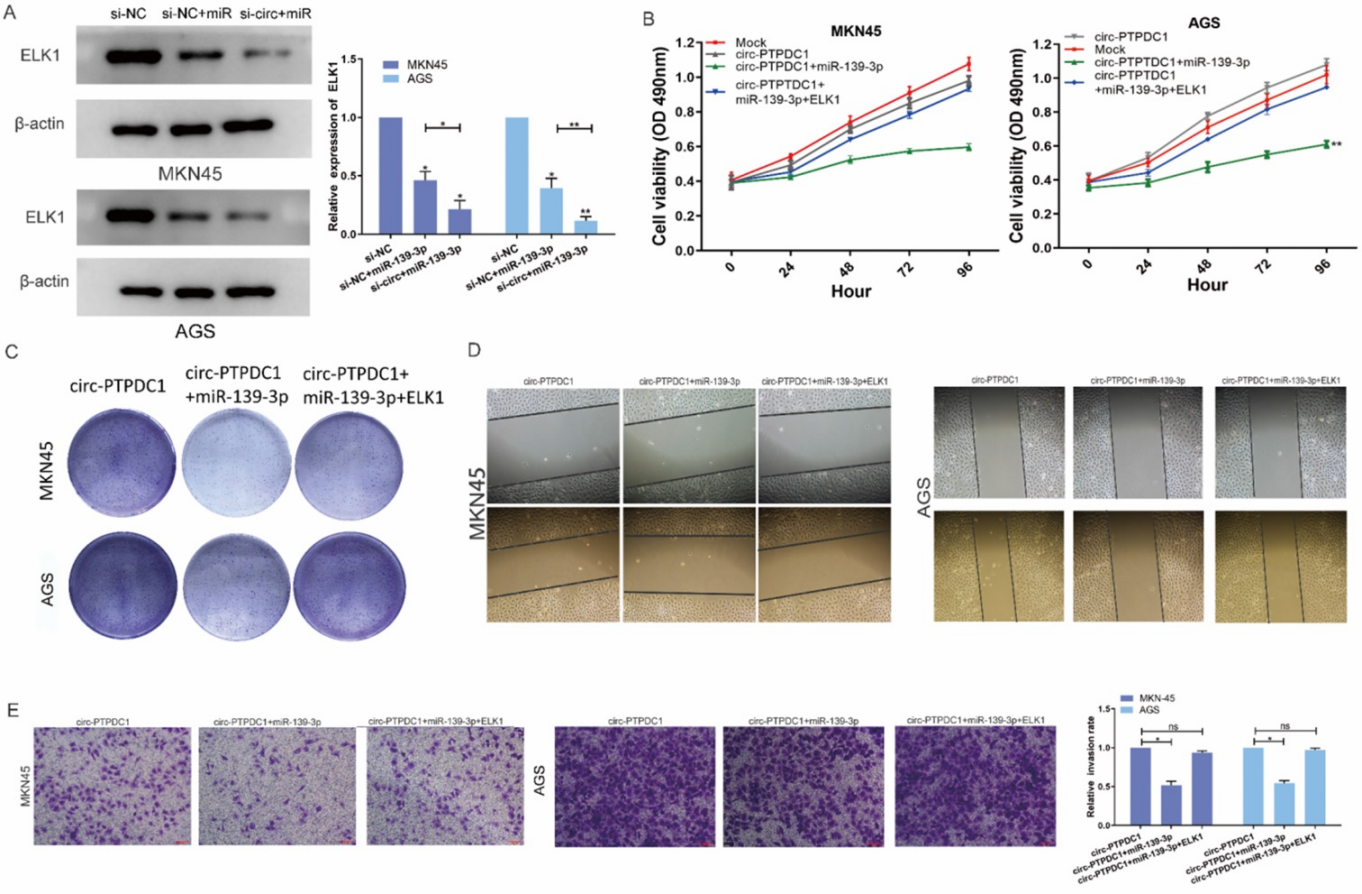
** circ-PTPDC1 promotes the proliferation, migration and invasion of GC by sponging miR-139-3p to regulate ELK1. (A)** The expression level of ELK1 treated with miR-139-3p mimics and si-circ-PTPDC1 +miR-139-3p mimics was significantly decreased compared with NC in GC cell lines. **(B-C)** The proliferation ability and colony-formation ability of GC cells were evaluated by CCK-8 and colony-formation assay which transfected or co-transfected with Mock, circ-PTPDC1 vector, miR-139-3p mimics and ELK1. **(D)** Cell motility was examined by wound healing assay in GC cells transfected or co-transfected with circ-PTPDC1, miR-139-3p mimics and ELK1. **(E)** Cell invasion assays were performed in GC cells transfected or co-transfected with circ-PTPDC1, miR-139-3p and ELK1 by using transwell chamber with Matrigel.

We next explored whether the circ-PTPDC1-miR-139-3p-ELK1 regulatory loop participated in the proliferation, migration and invasion of GC cells. Both mRNA and protein expression levels of ELK1 transfected with miR-139-3p were reduced and co-transfection of si-circ-PTPDC1 and miR-139-3p strengthened this effect **(Fig. 8A).** CCK-8 assays proved that the proliferation ability of cells treated with mock vector and circ-PTPDC1 has no significant difference, treated with miR-139-3p suppressed GC proliferation dramatically and these effects disappeared after co-transfected with ELK1 **(Fig. 8B)**. The colony-formation assay also showed the same trends results with CCK-8 assay **(Fig. 8C).** The scratch wound assay and transwell assay revealed that miR-139-3p suppressed the malignant behavior of circ-PTPDC1 on GC cell migration and invasion ability. However, after co-transfected of ELK1 reversed these effects **(Fig. 8D-E).** These results of experiments suggested that circ-PTPDC1 promotes the proliferation, invasion and migration of gastric cancer cells by sponging miR-139-3p to regulate ELK1.

### Circ-PTPDC1 promotes the upgrowth and metabasis of gastric cancer in vivo

**Figure 9:**
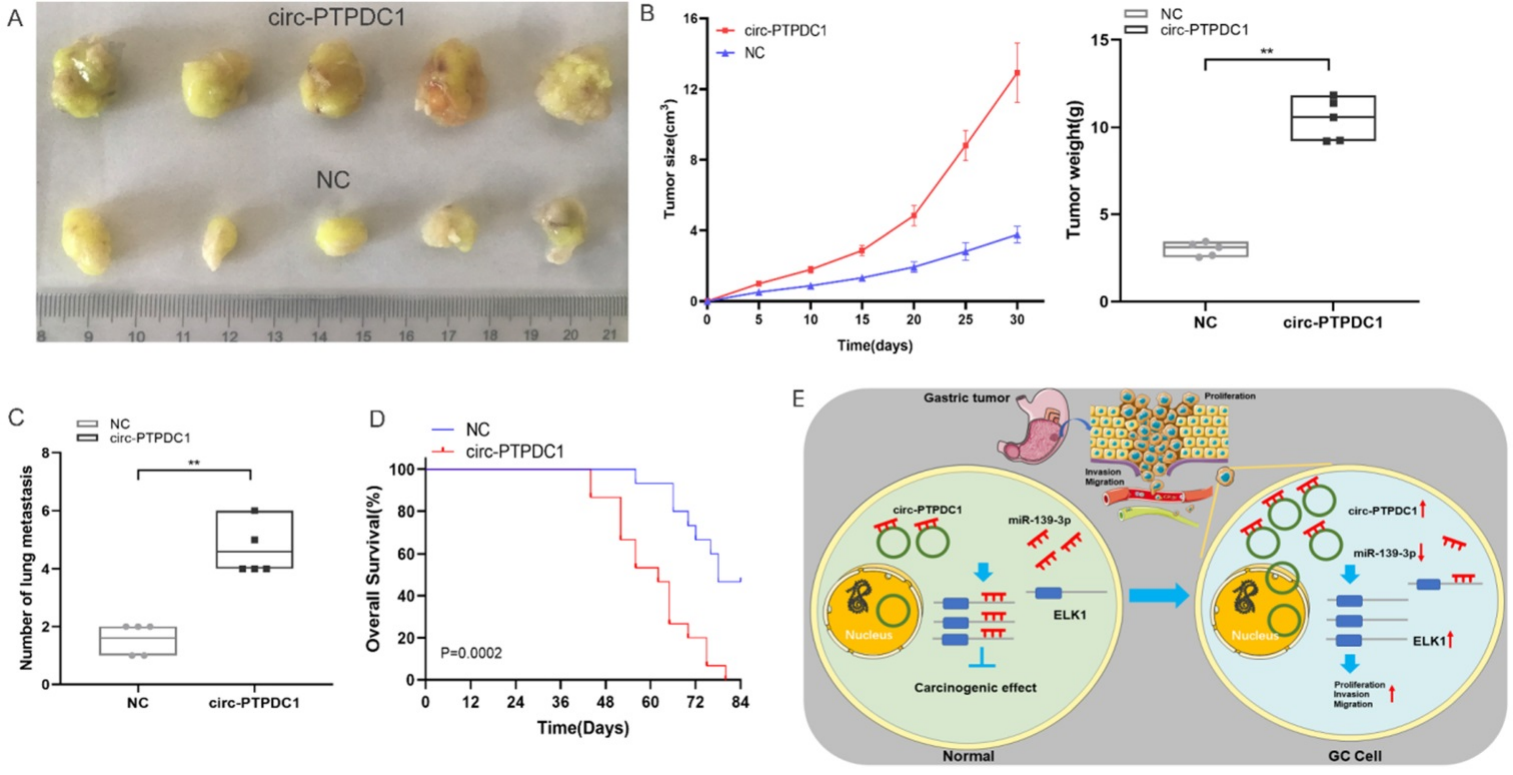
** Over-expression of circ-PTPDC1 inhibits the GC cells activities in vivo. (A)** Representative image of the xenograft GC tumors. **(B)** The upgrowth curves and weight of xenograft tumors. Xenograft tumor growth was measured by volume and tumor weight was recorded. **(C)** The number of lung metastasis in overexpression of circ-PTPDC1 and NC xenograft groups. **(D)** The OS of each group of mice injected with AGS cells. **(E)** The schematic diagram of the mechanism of circ-PTPDC1/miR-139-3p/ELK1 axis in GC. **p < 0.01.

To explore the association between circ-PTPDC1 and the upgrowth as well as metabasis of gastric cancer in vivo, MKN45 cells transfected with circ-PTPDC1 and NC was injected into nude mice to established xenograft tumor model and metabasis nude mice model **(Fig. 9A).** In the xenograft tumor model, we found that the overexpression of circ-PTPDC1 had a negative effect on the tumor size and weight of nude mice **(Fig. 9B).** Compared with the normally expressed circ-PTPDC1, the ectopic overexpression of circ-PTPDC1 inhibited metastasis in the lung **(Fig. 9C).** In addition, the circ-PTPDC1 group had a longer OS time than the NC group **(Fig. 9D).** Above all, these results illustrated that circ-PTPDC1 participated in the progression of GC through the crosstalk with ELK1 by competing for shared miR-139-3p** (Fig.9E).**

### The prognostic value of circ-PTPDC1

**Figure 10:**
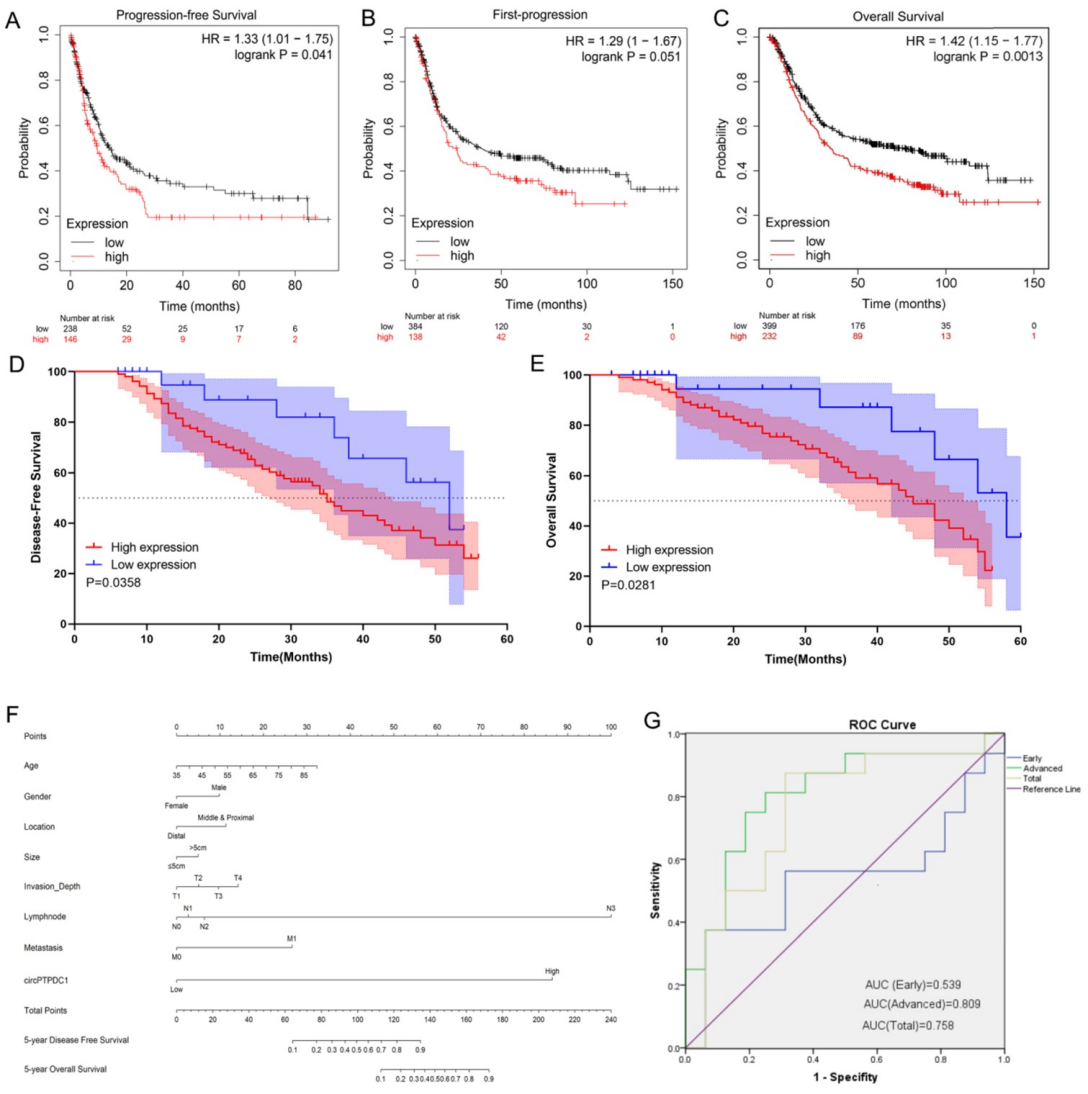
** circ-PTPDC1 could be a prognostic biomarker of GC. (A-C)** High expression of PTPDC1 was positively correlated with poor PFS, FP and OS in GC patients. **(D)** DFS of 128 GC patients based on the circ-PTPDC1 expression status (p =0.0358, log-rank). **(E)** OS of GC patients based on the circ-PTPDC1 expression status (p =0.0281, log-rank). **(F)** A predictive model determined by circ-PTPDC1 expression and independent prognostic factors to predict the probability of 5-year DFS and 5-year OS. **(G)** The ROC curve was used to evaluate the potential diagnostic value of circ-PTPDC1, the area under the ROC curve (AUC) in total being 0.758. The AUC of circ-PTPDC1 expression in advanced (III-IV) TNM stages of GC was 0.809, which is higher than that in early (I-II) TNM stages.

To delve into the prognostic potential of circ-PTPDC1 in GC, Kaplan-Meier Plotter was used. As presented in** Fig. 10A-C**, high expression of PTPDC1 was negatively correlated with PFS, FP and OS. To investigate the prognostic performance of circ-PTPDC1 expression in GC patients, survival curves were depicted in 128 patients according to prognostic information. Kaplan-Meier survival analysis and log-rank test of postoperative survival were performed to further evaluate the correlation between circ-PTPDC1 expression and prognosis in GC patients. The results revealed that patients with higher-level circ-PTPDC1 expression had significantly shorter DFS and OS than those with lower levels of circ-PTPDC1 expression **(Fig. 10D-E).** Afterwards, we established a predictive model based on significant parameters in multivariate analysis to forecast the probability of 5-year DFS and OS, and the predicted accuracy was evaluated by Harrell's c-index. The constructed nomogram was employed by adding the points on the highest scale of each independent indicator. The total point score was further identified on the total points scale to determine the probability of 5-year DFS and OS (c-index: 0.722; **Fig. 10F**). We further used the ROC curve to evaluate the diagnostic value of circ-PTPDC1 in distinguishing gastric cancer from adjacent noncancerous tissues. The area under the ROC curve (AUC) was 0.758, with a sensitivity of 87.5% and a specificity of 68.75%. We next focused on the correlation of circ-PTPDC1 with TNM stage and prognosis and then performed the ROC curve between the different TNM stages. Interestingly, the results showed that the AUC of circ-PTPDC1 expression in advanced (III-IV) TNM stages of GC was 0.809, higher than 0.539 in early (I-II) TNM stages **(Fig. 10G).** Taken together, dysregulated circ-PTPDC1 may serve as a key regulatory factor or prognostic biomarker of GC.

### Circ-PTPDC1 has a potentiality to encode protein

Circ-PTPDC1 contains an internal ribosome entry site (IRES) that can be efficiently translated. The IRES offers an optional cap-independent translation initiation site in eukaryotic cells **(Table S4).** As shown in **Fig. S3A**, we made a prediction of circ-PTPDC1 and fortunately found that circ-PTPDC1 had an m6A modification structure with great translation potential. ORF is another crucial element in the circular RNA sequence that shows its potential protein coding ability. Our prediction results revealed that circ-PTPDC1 had a structure of ORF **(Fig. S3B & Table S5).** In addition, circ-PTPDC1 can also interact with different RNA bind proteins and transcription factors **(Fig.S3C).** All the above findings suggest that circ-PTPDC1 may have the potentiality to encode proteins.

## Discussion

With the widespread use of high throughput circRNA microarray and the rapid development of bioinformatics, circRNAs have caught increasing attention and become a burgeoning research focus in recent years 27. Recently, circRNAs are gradually considered as a new type of endogenous non-coding RNAs (ncRNAs). Many circRNAs regulate protein expression by sponging miRNAs to further participate in cancer pathogenesis 28. Human circRNA cerebellar degeneration-related protein 1 transcript (CDR1) acts as a miR-7 sponge by binding with miRNA effector complexes. It is positively correlated with miRNA target site protein Argonaute (AGO) in a miR-7-dependent manner, and participates in inhibitng miR-7 activity and increasing miR-7 target gene expression level 29. Analogically, the sex-determining region Y (Sry) of testis-specific circRNA, functions as the sponge of miR-138 15. More studies have demonstrated that circRNAs play critical roles in various types of tumors and have great diagnostic and therapeutic potential for various types of cancers including GC 30-31.

Although increasing evidence shows that circRNAs play a regulatory role in various physiopathological regulation processes, the specific biological mechanism remains unclear. In this study, we used a human circRNA microarray to assess the differences in the circRNAs expression profiles of the tumor and non-tumor tissues from GC patients. Based on the bioinformatics analyses and the confirmation of *in-vitro* experiments, an observably upregulated circRNA known as circ-PTPDC1 was selected for further study. circ-PTPDC1 was traced from the 2-6 exons of PTPDC1, which was also up-regulated in GC. qRT-PCR showed that circ-PTPDC1 was significantly over-expressed in GC tissues and plasma compared with normal controls. Clinicopathological features illustrated that overexpression of circ-PTPDC1 was positively correlated with age, invasion depth and clinical stages. Additional univariate and multivariate analysis confirmed that circ-PTPDC1 overexpression, together with invasion depth and the clinical stage, was correlated with poor prognosis for GC patients in terms of OS and DFS.

The *in-vitro* experiments of current study proved that circ-PTPDC1 knockdown inhibited GC cell proliferation migration and invasion significantly, suggesting that circ-PTPDC1 may be closely associated with the development and progression of GC. Luciferase reporter assay revealed that the miR-139-3p mimics decreased the relative luciferase expression in circ-PTPDC1-WT group compared with the NC group. In addition, the expression level of miR-139-3p in the GC tumor tissues was significantly lower than that in the adjacent noncancerous tissues. A previous study 32 demonstrated that miR-139-3p was down-regulated in cervical cancer tissues and cell lines, demonstrating that miR-139-3p acted as a tumor suppressor that inhibited cervical cancer cell proliferation, migration and invasion and lead to cell apoptosis through down-regulating the NOB1 expression. Yonemori et al. 33 reported that miR-139-3p downregulation enhanced bladder cancer cell migration and invasion by targeting MMP11, showing that it was a good prognostic marker for survival of bladder cancer patients 33. In addition, Ng L and his colleagues 34 reported that serum miR-139-3p level in colorectal cancer patients was significantly lower than that in controls with a sensitivity of 96.6% and a specificity of 97.8%. All these studied suggested that miR-139-3p might have an inhibitory effect against cancers, which is consistent with the results of our study in GC. However, we have only studied one miRNA that binds to circ-PTPDC1, and more miRNAs, binding sites and expression regulation need to be further explored. Therefore, a network diagram was built to show all possible miRNAs and their target protein, which could be sponged by circ-PTPDC1.

To further elucidate the regulation mechanism, we looked into the target proteins regulated by miR-139-3p. ELK1 (ETS like transcription factor-1) is a member of the ternary complex factor (TCF) subfamily of ETS-domain transcription factors 35. Some *in-vitro* experiments showed that ELK1 activity may be critical for promoting proliferation and blocking apoptotic cell death in tumors 36. But it is unclear about the roles that ELK1 may play in GC. The result of the present study showed that ELK1 expression in GC tumor tissues was statistically higher than that in their adjacent noncancerous tissues, which is consistent with the results in TGCA database. Next, we explored whether circ-PTPDC1-miR-139-3p-ELK1 regulatory loop participated in the invasion and migration of GC cells. The results showed that the proliferation, invasion and migration ability of cells treated with miR-139-3p and si-circ-PTPDC1 + miR-139-3p was significantly suppressed compared with that treated with si-NC in GC cells. In addition, both mRNA and protein expression levels of ELK1 treated with miR-139-3p or si-circ-PTPDC1 + miR-139-3p were significantly decreased compared with the control group in AGS and MKN45 cell lines. We also found that miR-139-3p suppressed the malignant behavior of circ-PTPDC1 on GC cell proliferation, migration and invasion ability, nevertheless, after the co-transfection of ELK1 reversed these effects. In summary, these results reflect that circ-PTPDC1 can be identified as a sponge of miR-139-3p which can promote the proliferation, migration and invasion of GC cells by regulating functionally targeted ELK1 through down-regulating miR-139-3p.

The online database Kaplan-Meier Plotter shows that high expression of PTPDC1 is correlated with shorter PFS, FS and OS in GC patients. These results are compatible with the findings of the present study. Based on our follow-up data, Kaplan-Meier survival curve was conducted, showing that GC patients with higher circ-PTPDC1 expression had shorter DFS and OS. Then, we established a prognostic prediction model in the form of a nomogram and found that circ-PTPDC1 played an essential role in GC carcinogenesis.

circRNA is normally regarded as a non-coding RNA 37. However, most circRNAs may also have the potential to translate protein production since they are derived from exons and located in the cytoplasm 38. N6-methyladenosine (m6A) is the most abundant RNA internal modification in eukaryotes and occurs preferentially in the common motif-rRM6ach (R = G or A; H = A, C or U). Previous studies 39 have demonstrated that m6A-driven circRNA translation exists widely, and hundreds of endogenous circRNAs have translation potential. If conservative sequences are present in circRNAs, protein translation can be initiated by this mechanism. In addition, IRES and ORF can be added to translate engineered circRNAs, and some circRNAs can be transcribed 40. It was found in the present study that circ-PTPDC1 m6A had the structure of m6A modified ORF and IRES, indicating that circ-PTPDC1 has the potential to encode proteins. If such translation products exist endogenously, they may play a specific biological role or interfere with protein-protein interactions. However, it is not clear whether and how these circRNAs are translated into proteins in normal conditions. With the discovery of more biological functions in circRNAs, this newly named circRNA known as circ-PTPDC1 would be applied more widely in GC.

An increasing number of studies has concentrated on the relationship between circRNAs and the development of GC from the clinical perspective, and the results have demonstrated that circRNAs can be used as clinical tumor biomarkers. However, most of these studies only detected the expression of circRNAs in cancer and adjacent noncancerous tissue, and few studies have compared the circRNA expression between pre- and postoperative plasma samples. In addition, the sample size in these studies is relatively small. By comparison, more samples were included in our study and we have conducted a relatively sufficient follow-up of the patients in our study. Based on our follow-up data, a prognostic model was established in the form of a nomogram. This model can show the contribution of circ-PTPDC1 with other classic clinical factors together intuitively and visually in the prognosis of GC patients. The results suggest that circ-PTPDC1 may prove to be an ideal non-invasive biomarker for the diagnosis and prognostic prediction of GC. To the best of our knowledge, this is the first study to investigate the role of circ-PTPDC1 in GC. In addition, this is also the first article to elucidate the relationship between miR-139-3p and ELK1. The findings may have implications for the treatment of GC.

There are some limitations in interpreting the results of our research. Firstly, our study used GC samples from an ethnically homogenous population, and we expect more abundant sample size and more validation from different regions. Secondly, our study examined the ability of circ-PTPDC1 to bind to miR-139-3p, but other miRNAs that can bind circ-PTPDC1 to regulate the occurrence and progression of GC may exist. Finally, it requires further investigation on whether circ-PTPDC1 modulates the biological behavior of GC through other mechanisms, such as protein binding. We hope that a follow-up study will illuminate a deeper understanding of the therapeutic potential of circ-PTPDC1.

## Conclusion

Our study identifies a new circular RNA, termed circ-PTPDC1 that is up-regulated in tumor tissues, cells and plasma of GC patients, and can act as a sponge of miR-139-3p to regulate the expression of ELK1. Our findings suggest that circ-PTPDC1 may be a novel potential circulating biomarker for the detection of GC.

## Material and Methods

### Patients and samples

Among the GC patients who underwent surgery in Nanjing First Hospital from 2013 to 2017, amount to 128 pairs of GC and corresponding adjacent noncancerous tissues were obtained (Nanjing, China). Thereinto, 6 pairs of tissues were selected to be applied in the Arraystar Human Circular RNA Microarray V2.0 (Arraystar, Inc., Rockville, MD, USA). Peripheral blood (5ml) of the 128 GC patients was obtained before operation and separated for plasma by centrifugation. Fresh plasma samples from 62 healthy people were used as control. Pathological analysis confirmed that the adjacent noncancerous tissues were matched with no tumor cells at the margins, and they were located 5 cm away from the edge of the GC site. After the tissue samples were obtained, they were immediately stored at -80 °C until use. None of the patients received radiotherapy or chemotherapy before operation. According to the tumor-node-metastasis (TNM) staging system of the International Union against Cancer (v.8; 2016), all tumors were staged accurately. Histopathological diagnosis of these specimens was confirmed and classified by two experienced clinical pathologists.

### The design and analysis of high throughput microarray

#### CircRNA microarray

The Arraystar Human Circular RNA Microarray V2.0 (Arraystar, Inc., Rockville, MD, USA) was designed for global profiling of human CircRNAs. Genesspring software V13.0 (Agilent) was used to analyze the circRNA array data for data aggregation, normalization and quality control. The threshold values of logFC ≥1 and ≤-1 changes and a Benjamini-Hochberg corrected *p*-value were used to choose the differentially expressed genes. Log2 transformation of data was conducted using CLUSTER 3.0 software to adjust the data function, and gene-centered midranking was performed, followed by hierarchical clustering and average linkage analysis.

### Bioinformatics analysis and data mining

To figure out the participation of the circRNA array data in the biological process and their connection of tumorigenesis, we conducted a Kyoto Encyclopedia of Genes and Genomes (KEGG) analysis and Gene Set Enrichment Analysis (GSEA) using the R 4.0.2 software (Institute for Statistics and Mathematics, Vienna, Austria) with the R package “clusterProfiler” 18-19. Weighted correlation network analysis (WGCNA) was accomplished with the R package “WGCNA” 20. The circRNA array data were applied as the input. The predicted gene functions of the top 50 cirRNAs with the largest expression differences were annotated using GO (http:www.geneontology.org) and the KEGG (https://www.genome.jp/kegg/) with the Database for Annotation, Visualization and Integrated Discovery (DAVID; https:david.ncifcrf.gov) 21. In addition, visualized enrichment networks of the target gene of these circRNAs were made by Metascape (http://metascape.org/gp/index.html#/main/step1) 22. To detect the expression of PTPDC1, the Cancer Genome Atlas (TCGA) (http://cancergenome.nih.gov) database was used to investigate the expression of PTPDC1 in different tumor tissues and corresponding para-carcinoma tissues. UALCAN (http:/ualcan.path.uab.edu/) is a comprehensive and interactive website to analyze cancer OMICS data 23. It was used here to conduct subgroup analysis of PTPDC1 expression in GC. The Human Protein Atlas (https://www.proteinatlas.org/) database contains pathology and gene information from many reports on various tissues and cells. We used it to confirm the intracellular location of PTPDC1 mRNA and ELK1 staining in stomach normal tissues and GC tissues. Evidence of the Ago2 binding site was obtained from the public online cross-linked immunoprecipitation (CLIP) data set, which is available from doRiNA (http://dorina.mdc-berlin.de). Circrnainteractome (https://circinteractome.nia.nih.gov/), starBase v3.0 (http://starbase.sysu.edu.cn/index.php) and Targetscan (http://www.targetscan.org/vert_72/) databases were used to ascertain the potential target miRNAs that could be sponged by circ-PTPDC1 24-25. MIRDB database (http://www.mirdb.org/) was used to identify the potential miR-139-3p's target protein 26. GEPIA2 database (http://gepia2.cancer-pku.cn/#index) was used to detect the expression level of ELK1 in stomach adenocarcinoma (STAD).

### *In-vitro* experiments

#### Cell line, cell culture, and transfection

Human GC cell lines AGS, MKN28, SGC-7901, MKN-45 and MGC-803 were constrcuted from specimens extracted from human GC patients by Shanghai Institutes for Biological Sciences of the Chinese Academy of Sciences (Shanghai, China). We acquired the human gastric epithelial cell line GES-1 from the Cancer Institute and Hospital of the Chinese Academy of Medical Sciences (Beijing, China). Si-circ-PTPDC1, miRNA-139-3p mimic, miRNA-139-3p inhibitor and their related control oligonucleotide were designed and synthesized by RiboBio (Guangzhou, China). The target sequence for circ-PTPDC1 siRNAs was as follows: siRNA-1: 5'-GAACAGACTACCATGGCTG-3'; siRNA-2: 5'-TGGTCGAACAGACTACCAT-3'; siRNA-3: 5'-GGCTTCCTCGAACAGACTA-3'. All transfection procedures were performed by the final concentration of 60 nM of miRNA mimics and 100 nM of miRNA inhibitor and si-circ-PTPDC1 or a scrambled negative control (si-NC). Lipofectamine 2000 transfection reagent (Invitrogen, Carlsbad, CA, USA) was used as transfection medium. After 48 h, knockdown of circ-PTPDC1 was confirmed via quantitative real-time Polymerase chain reaction (qRT-PCR) applying the ABI7500 System (Applied Biosystems, Foster City, CA, USA).

### RNA isolation, reverse transcription and qRT-PCR

Total RNA from the paired tissues was extracted through TRIzol reagent (Thermo Fisher Scientific, Waltham, MA, USA). Total RNA in plasma was extracted by TIANamp Virus RNA Kit according to producer's protocol. cDNA was synthesized by using reverse transcription kit (Takara, Otsu, Japan) for circRNA and mRNA. Total RNAs were reversed using the RiboBio reverse transcription kit (Guangzhou, China) for miRNA. The mRNA and circRNAs were quantified using SYBR GreenPCR kit (Takara, OTSU, Japan), and SYBR Green PCR Kit (RiboBio, Guangzhou, China) was used for miRNA PCR. The circ-PTPDC1 expression level was determined by qRT-PCR using the following primer pair: 5′-CTTTCATGGAGGCTGGCATT-3′ (Forward, or F) and 5′-TGCAGCCATGGTAGTCTGTT-3' (reverse, R). Glyceraldehyde 3-phosphate dehydrogenase (GAPDH) was used as an internal control, with a primer pair 5'-GCATCCTGGGCTACACTG-3' (F) and 5'-ACTTCAGGAGCATCTGAAATAGGT-3' (R). All qRT-PCR reactions were carried out employing the ABI7500 System (Applied Biosystems, Foster City, CA, USA). The relative expression fold change of mRNAs was calculated by the 2^-ΔΔCt^ method. Each sample was made in triplicate, and all experiments were repeated independently for three times to ensure the repeatability of all data.

### Sanger sequencing and RNase R treatment

The amplification products were interposed into a T-vector for Sanger sequencing to ascertain their full-length. The divergent primers were designed to confirm the back-splice junction of circ-PTPDC1: 5'-AGGGCTTGGTCGAACAG-3' (Forward) and 5'-ACTACCATGGCTGCAGG-3' (Reverse). The primers were synthesized by Invitrogen (Shanghai, China), and Sanger sequencing was performed by Realgene (Nanjing, China). A total of 2 mg RNA was incubated for 20 min at 37 °C with or without 3 units/mg of RNase R. RNeasy MinElute cleaning Kit (Qiagen) was used to purify the resulting RNA. To assess the stability of circ-PTPDC1 and linear PTPDC1 mRNA, the expression levels were determined by qRT-PCR.

### Isolation of cytoplasmic and nuclear RNA

A total of 1×10^6^ GC cells were collected and washed twice in phosphate buffered saline (PBS). The suspension was centrifuged at 4 °C, 500 rpm for 5 min. The supernatant was removed and cleaned twice. Cytoplasmic and nuclear RNA were separated and purified using the PARIS Kit (Life Technologies, Carlsbad, CA, USA) in accordance with the producer's explanation.

### Cell Counting Kit-8 (CCK-8) proliferation assay

Cell proliferation was determined using the Cell Counting Kit-8 (CCK-8, Promega) assay according to the manufacturer's instructions. MKN45 and AGS cells were transfected with si-NC or 100 nM si-circ-PTPDC1 and seeded in 96-well plates at 3×10^3^ per well and monitored. An amount of 10 μl CCK-8 reagent was added to each well, and the cells were hatched for two hours in a 37 °C cell incubator. A microplate reader (MD, USA) at an absorbance of 450 nm was used to evaluate the cell viability every 24 h. Each experiment was repeated in triplicate.

### Colony formation experiment

Plant the gastric cancer cell lines MKN-45 and AGS transfected with si-circ-PTPDC1 in a six-well plate, add complete medium containing 10% fetal bovine serum, then, place it in a 37 °C cell incubator containing 5% CO2 Cultivate for 10 days, and replace the complete medium every 3 days. After completing the culture, remove the medium, wash the cells with PBS, fix the cells with 4% paraformaldehyde for 15 minutes, discard the paraformaldehyde, stain with 0.1% crystal violet dye for 20 minutes, and wash the background with PBS.

### Scratch wound assay

After planting the gastric cancer cell lines MKN-45 and AGS in a 6-well plate, the cell line was transfected with si-circ-PTPDC1, and the cells were cultured until the confluence reached 100%. Artificial linear wound on layer of cells, use PBS to remove free-floating cells and debris produced by the wound. Add complete medium and incubate in a 37 °C cell incubator. Take pictures using an inverted microscope at 0 and 24 hours. Measure the width of the original wound and the width of the wound after cell migration, and the experiment repeated 3 times.

### Cell migration and invasion assays

Add 200 µl of serum-free RPMI 1640 medium to the upper chamber of Transwell (Millipore, Billerica, MA, USA), and inoculate the MKN-45 and AGS cell lines transfected with si-circ-PTPDC1. The Matrigel mixture (BD Biosciences, San Jose, CA, USA) was spread over the Transwell chamber for the invasion test. Matrigel is not required for migration test. Add 700 µl of RPMI 1640 medium containing 10% FBS to the lower chamber of the Transwell, insert it into the upper chamber of the Transwell, and incubate it in the cell culture incubator for 24 hours. Use a sterile cotton swab to wipe off the remaining cells and Matrigel in the upper chamber of the Transwell. Use 4% Paraformaldehyde was fixed and stained with 0.1% crystal violet dye. The cells were photographed and counted in five fields of view.

### RNA immunoprecipitation (RIP)

Perform RIP analysis according to the instructions of EZ-magna RNA Immunoprecipitation Kit (Millipore). The whole-cell extract was then incubated with RIP buffer containing magnetic beads conjugated to anti-AGO2 or control IgG. The beads were washed using a washing buffer, followed by the addition of proteinase K to digest the proteins. Use RIP lysis buffer to lyse the collected cells, and then separate the precipitated RNA for qRT-PCR analysis.

### Luciferase reporter assay

Pre-designed and synthesized the wild-type and mutant fragments of circ-PTPDC1 and ELK1, and inserted them into the pGL3 promoter vector (Realgene, Nanjing, China). MKN45 and AGS cells were seeded on a 96-well plate and cultured in a medium containing 10% FBS, and incubated in a 37 °C, 5% CO2 incubator. They were co-transfected with luciferase reporters and miR-139-3p mimics. After 48 hours of incubation, the fluorescein in the cells was collected according to the instructions Enzyme Mars and detected by the dual luciferase reporter gene detection system (Promega, Madison, Wisconsin, USA).

### Biotin-coupled probe RNA pull-down assay

To detect the miRNA sponged by circRNA, biotin-coupled probe RNA pull-down assay was performed. MKN45 and AGS cells transfected with miR-139-3p mimics were lysed and incubated with the biotin-coupled probe of circ-PTPDC1, which was pre-bound on magnetic beads. For approximately two hours, the target RNA was pulled down by the RNeasy Mini Kit (QIAGEN, Germany). Subsequently the pull-down product was extracted, reversed and quantitated using qRT-PCR. Each experiment was repeated three times to ensure the credibility of results.

### Western blot assay

Cells were lysed using RIPA lysis buffer (RIPA, Beyotime, China) supplemented with protease inhibitor cocktail (Pierce Biotechnology). The protein was prepared with protein concentrations and determined using the BCA Protein Assay Kit (Pierce Biotechnology). The same amount of protein was extracted by 10% SDS-PAGE and transferred onto a PVDF membrane (Millipore, Schwalbach, Germany). Use 5% skimmed milk powder blocking solution to incubate with ELK1 primary antibody (1:1000, Abcam, USA). Anti-β-ACTIN antibody (1:1000, Abcam, USA) at 4 °C for 12 h. Then the prepared membrane was incubated with secondary antibody (1:5000) for 2 h. Finally, use ECL Luminescence Kit (Amersham) to detect western blot, and use Image Lab Software for relevant data analysis.

### *In-vivo* tumor xenografts

All animal experimental procedures and animal care were in accordance with the guidelines provided by the National Institutes of Health (NIH). The Animal Experiment Ethics Committee of Nanjing Medical University has approved the experiments and projects related to experimental animals. To create the xenograft tumor model, 20 five-week-old male BALB/c nude mice were separated randomly into over-circ-PTPDC1 group and NC group (n = 10 for each group). The circ-PTPDC1 group was subcutaneously injected with AGS cells transduced with the circ-PTPDC1 vector, and the NC group was subcutaneously injected with AGS cells transduced with NC vector. The volume of all injected nude mice was measured every three days by using digital calipers. One-month later, all injected nude mice were sacrificed, and xenograft tumors were resected, dissected and weighted. Tumor volume was calculated according to the following equation: Volume (cm^3^) = 0.5 × Length (cm) × Width^2^ (cm^2^). For metastasis model, AGS cells were injected via the tail vein (15 mice per group). After six weeks, five mice of each group were sacrificed. The lungs were fixed in 4% paraformaldehyde and stained with hematoxylin and eosin (HE). Lung metastatic foci were counted microscopically. The remaining mice were observed for survival analysis with 12 weeks as cutoff.

### Follow-up and establishment of prognostic model

Kaplan-Meier Plotter (http:/kmplot.com/analysis), an online survival analysis tool, was used to analyze the correlations between PTPDC1 expression and progression-free survival (PFS), first-progression survival (FPS) and OS. To document follow-up evaluation, all patients were advised to visit their doctors every three months in the first year, every six months in the second year and annually thereafter until December 2019. The attending doctors conducted monthly telephone follow-up interviews with the patients until June 2020. We use telephone follow-up interviews, hospitalization and outpatient records to obtain the specific date of relapse or death. The Kaplan-Meier method with the log-rank test was applied to assess the relationship between circ-PTPDC1 expression, DFS and OS. Receiver operating characteristic (ROC) curves were constructed to evaluate the diagnostic value of circ-PTPDC1 levels, by plotting sensitivity versus 100% specificity. Based on the medical records of the follow-up patients, a five-year DFS and five-year OS nomogram was established by using R 4.0.2 software (Institute for Statistics and Mathematics, Vienna, Austria). The expression of circ-PTPDC1 and gender, age, tumor size, tumor location, differentiation, clinical stage, depth of invasion, lymph node metastasis, OS, DFS description are all in the nomogram, and use the Harrell consistency index (c-index) To evaluate the accuracy of the forecast.

### Statistical analysis

The significance of the differences between groups was estimated by the Student's t-test, χ^2^ test or Wilcoxon-test, as appropriate. A chi-square test or Fisher's exact test was conducted to analyze categorical data. The paired t-test was used to test the significance of the difference between two pairs of paired data, and the independent sample t-test or Mann-Whitney *U* test was selected to examine the difference in continuous variables. Continuous data were expressed as the mean ± standard deviation (SD). The total difference between the three groups was measured by the one-way analysis of variance. Survival data were evaluated using univariate and multivariate Cox proportional hazards models. Variables with a value of *p*<0.05 in univariate analysis were used in the ensuing multivariate analysis based on Cox regression analyses. The statistical analyses were mainly performed by using SPSS (Version 23.0, IBM, USA). *P*-value less than 0.05 was demarcated to be statistically significant.

### Annotation of some potential functions of circ-PTPDC1

The IRES was annotated according to IRESite (http://iresite.org/IRESite_web.php). The open reading frame (ORF) was predicted in line with ORF Finder (https://www.ncbi.nlm.nih.gov/orffinder). N6-methyladenosine (m6A) modification preferably occurs in the consensus motif “RRm6 ACH” (R = G or A; H = A, C or U). The interaction network of circ-PTPDC and RBP, TF was made in RNA interactcome database (http://www.rna-society.org/rnainter/).

## Supplementary Material

Supplementary figures and tables.

## Figures and Tables

**Table 1:** The correlation between the circPTPDC1 expression and clinicopathological characteristics in GC patients

Clinical Information	Cases (n=128)	Percentage	circPTPDC1		χ^2^	P-value
			High Exp. (n=103)	Low Exp. (n=25)		
**Age (years)**						
≤60	31	24.22%	19	12	28.733	<0.001*
>60	97	75.78%	84	13		
**Gender**						
Male	86	67.19%	71	15	0.728	0.394
Female	42	32.71%	32	10		
**Location**						
Distal	47	36.72%	39	8	0.298	0.585
Middle & Proximal	81	63.28%	64	17		
**Size**						
≤5 cm	62	48.44%	49	13	0.158	0.691
>5 cm	66	51.56%	54	12		
**Histologic Differentiation**						
Well & Moderately	22	9.38%	19	6	0.17	0.68
Poorly & Undifferentiated	106	90.72%	87	19		
**T Stages**						
T1-T2	38	29.69%	25	13	7.41	0.006*
T3-T4	90	70.31%	78	12		
**pN Stages**						
pN0	38	29.69%	29	9	0.593	0.441
pN1-N3	90	70.31%	74	16		
**M Stages**						
M0	117	91.41%	99	18	1.81	0.179
M1	11	8.59%	4	7		
**Clinical Stages**						
I-II	76	59.38%	55	21	9.687	0.002*
III-IV	52	38.28%	49	3		

**Table 2:** Univariate and multivariate analysis for overall survival and disease-free survival

Variables	DFS			OS		
	HR	95%CI	p value	HR	95%CI	p value
**Univariate analysis**						
Age (<60 years vs. >60 years)	1.618	0.980-1.268	0.052	1.709	1.027-2.844	0.039*
Gender (male vs. female)	0.879	0.621-1.948	0.576	1.744	0.604-1.495	0.708
Location (Distal vs. Middle+Proximal)	0.794	0.610-1.214	0.247	0.813	0.741-1.832	0.816
Tumor size ( >5 cm vs.<5 cm)	0.926	0.806-1.452	0.801	0.918	0.791-1.709	0.987
Differentiation (Well+ Moderately vs. Poorly+ Undifferentiated)	1.921	0.944-2.167	0.211	1.781	0.892-2.197	0.352
Invasion depth (T3+T4 vs. T1+T2)	1.518	1.021-2.111	0.013*	2.455	1.654-4.126	0.005*
TNM stage (III + IV vs. I+II)	1.379	1.122-1.689	0.006*	1.613	1.451-2.222	0.001*
Lymphatic metastasis (No vs. Yes)	0.823	0.633-1.013	0.143	0.834	0.621-1.646	0.087
Regional lymph nodes (PN2+ PN3 vs. PN0+ PN1)	0.831	0.912-2.245	0.219	0.857	1.254-3.012	0.101
Distant metastasis (No vs. Yes)	0.493	0.302-0.890	0.244	0.518	0.220-0.903	0.118
Expression of circ-PTPDC1 (High vs. Low)	2.296	1.679-3.742	0.0001*	2.688	1.793-4.761	<0.0001*
**Multivariate analysis**						
TNM stage (I+II vs. III + IV)	0.769	0.812-1.643	0.017*	0.788	0.655-1.218	0.039*
Invasion depth (T3+T4 vs. T1+T2)				0.852	0.795-1.809	0.032*
Regional lymph nodes (PN0+ PN1vs. PN2+ PN3)				0.779	0.363-1.674	0.522
Distant metastasis (No vs. Yes)	0.614	0.431-1.268	0.639	0.469	0.087-0.894	0.101
Expression of circ-PTPDC1 (High vs. Low)	1.264	1.112-1.825	0.048*	1.835	0.997-3.475	0.042*

HR, hazard ratio; 95% CI, 95 % confidence interval. *P<0.05.

## Abbreviations

CCK8: Cell counting kit-8
CeRNA: competitive endogenous RNA
CircRNAs: Circular RNAs
ELK1: E26 transformation-specific Like-1 protein
FBS: fetal bovine serum
GAPDH: Glyceraldehyde-3-phosphate dehydrogenase
GC: Gastric cancer
GO: Gene oncology
GSEA: Gene Set Enrichment Analysis
HE: Hematoxylin and Eosin
KEGG: Kyoto Encyclopedia of Genes and Genomes
LATS1: Large tumor suppressor kinase 1
m6A: N6-methyladenosine
RBP: RNA-binding protein
miRNAs: MicroRNAs
Mut: mutant
NIH: National Institutes of Health
NC: Negative control
ORF: Open reading frame
OS: Overall survival
PBS: phosphate buffered saline
PFS: Progression-free survival
FPS: First-progression survival
PTEN: Phosphatase and Tensin Homolog
qRT-PCR: Quantitative reverse transcription polymerase reaction
RIP: RNA immunoprecipitation
RIPA: Radioimmunoprecipitation assay
ROC: Receiver-operating characteristic
SD: standard deviation
TF: Transcription factor
TGCA: the Cancer Genome Atlas
WGCNA: Weighted correlation network analysis
WT: Wild type
