# Supplementary material for: Circ-PTPDC1 promotes the Progression of Gastric Cancer through Sponging Mir-139-3p by Regulating ELK1 and Functions as a Prognostic Biomarker
Source: Int J Biol Sci. 2021 Oct 17;17(15):4285–304. doi: 10.7150/ijbs.62732 (PMC8579456; doi:10.7150/ijbs.62732)
Supplement: Supplementary file 1 — Supplementary figures and tables. [file ijbsv17p4285s1.pdf]

Supplementary Material

Figure S1. A total of 10 circRNAs based on the multiple fold difference between the expression of GC plasmas and normal controls were verified that in a small sample of plasmas by using qRT-PCR. *P<0.05, **P<0.01,***P<0.001.

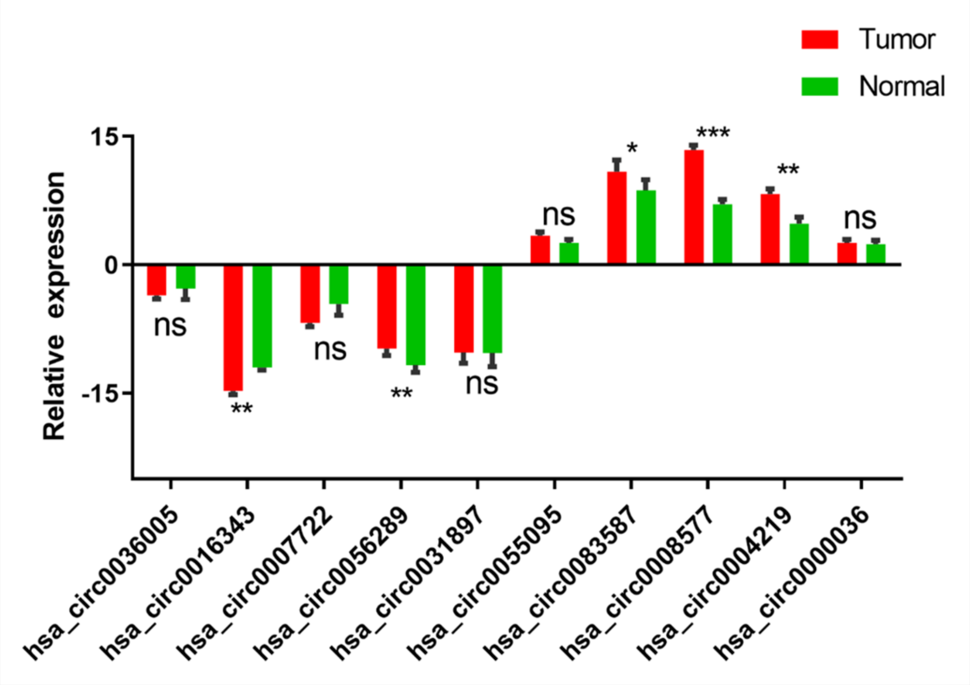

Figure S2. Identification of miR-139-3p as a target of circ-PTPC1 using circInteractome, starBase and TargetScan.

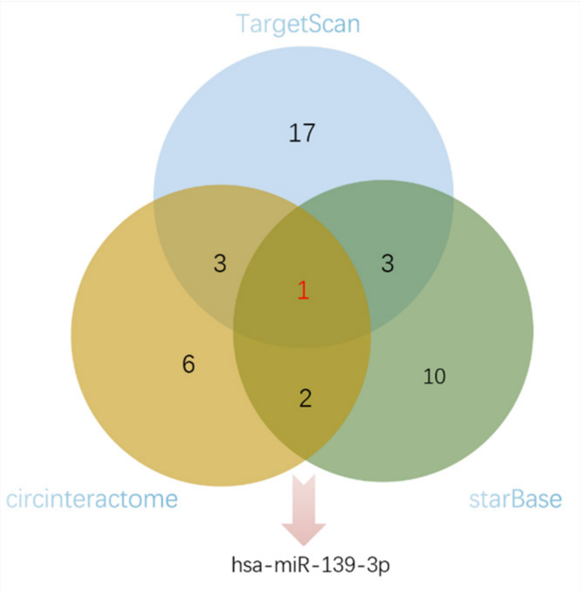

Figure S3. circPTPDC1 serves as a sponge for multiple miRNAs and has potential ability to encode protein. (A) circPTPDC1 has the structure of m6A modification-RRm6ACH (R = G or A; H = A, C or U). **(B)** CircPTPDC1 has the structure of ORF.**(C)** Transcriptions factors and RNA-binding proteins associated with CircPTPDC1

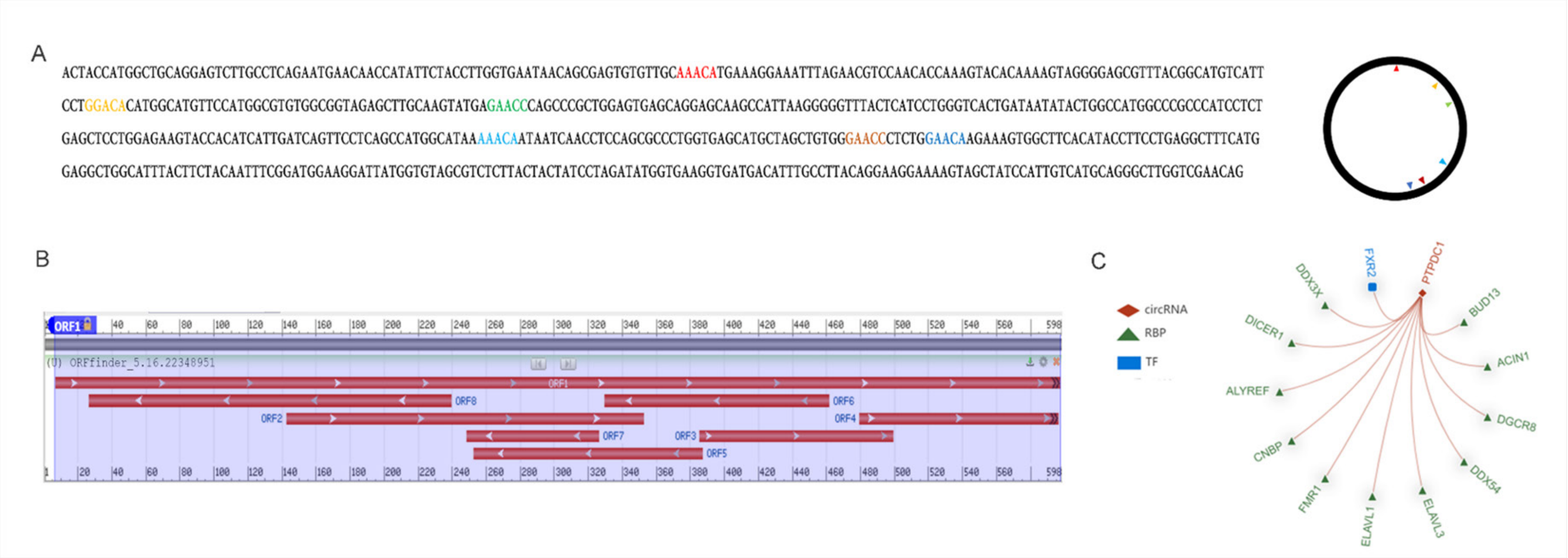

Table S1. The clinicopathological features of 6 samples used for circularRNA Microarray

ID	Age	Gender	Location	Size	Differentiation	Invasion Depth	Lymphnode	Metastasis	Clinical Stage
1	45	Male	Middle & Proximal	≤5cm	Undifferentiated	T4	N1	M0	4
2	64	Male	Middle & Proximal	≤5cm	Poor	T3	N2	M1	3
3	71	Male	Distal	>5cm	Moderately	T2	N0	M1	1
4	58	Female	Middle & Proximal	>5cm	Well	T1	N0	M1	1
5	76	Female	Distal	>5cm	Undifferentiated	T3	N3	M0	4
6	80	Female	Middle & Proximal	>5cm	Poor	T3	N0	M1	2

Table S2. The subspecies details of the 128 gastric cancer patients.

Pathological types	Amount
Papillary	42
Tubular	33
Mucinous	26
Signet ring	15
Undifferentiation	12

Table S3. The top five target of miR-139-3p

Name	Score
ELK1	95
NOX4	93
ANXA2R	92
PLD5	88
PLCH1	87

Table S4. Circ-PTPDC1 internal ribosomal entry sites.

Sequences producing significant alignments:	Score(bits)	E-Value
IRESite_Id:77	26	2
IRESite_Id:109	23	22
IRESite_Id:111	23	22
IRESite_Id:474	23	22
IRESite_Id:71	23	22
IRESite_Id:57	23	22
IRESite_Id:366	23	22
IRESite_Id:314.	23	22
IRESite_Id:315	23	22
IRESite_Id:563	23	22
IRESite_Id:244	23	22
IRESite_Id:632	23	22
IRESite_Id:553	23	22
IRESite_Id:65	21	74
IRESite_Id:626	21	74
IRESite_Id:597	21	74
IRESite_Id:102	21	74
IRESite_Id:612	21	74
IRESite_Id:243	21	74
IRESite_Id:124	21	74
IRESite_Id:85	21	74
IRESite_Id:84	21	74
IRESite_Id:82	21	74
IRESite_Id:83	21	74
IRESite_Id:69	21	74
IRESite_Id:519	21	74
IRESite_Id:117	21	74
IRESite_Id:342	21	74
IRESite_Id:506	21	74
IRESite_Id:367	21	74
IRESite_Id:215	21	74
IRESite_Id:214	21	74
IRESite_Id:343	21	74
IRESite_Id:463	21	74
IRESite_Id:431	21	74
IRESite_Id:462	21	74
IRESite_Id:345	21	74
IRESite_Id:439	21	74
IRESite_Id:461	21	74
IRESite_Id:459	21	74
IRESite_Id:247	21	74
IRESite_Id:251	21	74
IRESite_Id:594	21	74
IRESite_Id:630	21	74
IRESite_Id:317	21	74

Table S5. The ORF structures in circ-PTPDC1

Label	Strand	Frame	Start	Stop	Length (nt | aa)
**ORF1**	**+**	**1**	**7**	**>597**	**591| 196**
ORF2	+	2	143	352	210 | 69
ORF3	+	2	386	499	114 | 37
ORF4	+	3	480	>596	117 | 38
ORF5	-	2	387	253	135 | 44
ORF6	-	3	461	330	132 | 43
ORF7	-	3	326	249	78 | 25
ORF8	-	3	239	27	213 | 70
